# Human iPSC co-culture model to investigate the interaction between microglia and motor neurons

**DOI:** 10.1038/s41598-022-16896-8

**Published:** 2022-07-23

**Authors:** Björn F. Vahsen, Elizabeth Gray, Ana Candalija, Kaitlyn M. L. Cramb, Jakub Scaber, Ruxandra Dafinca, Antigoni Katsikoudi, Yinyan Xu, Lucy Farrimond, Richard Wade-Martins, William S. James, Martin R. Turner, Sally A. Cowley, Kevin Talbot

**Affiliations:** 1 Oxford Motor Neuron Disease Centre, Nuffield Department of Clinical Neurosciences, John Radcliffe Hospital, University of Oxford, Oxford, OX3 9DU UK; 2 Kavli Institute for Nanoscience Discovery, University of Oxford, Dorothy Crowfoot Hodgkin Building, South Parks Road, Oxford, OX1 3QU UK; 3 Oxford Parkinson’s Disease Centre, Department of Physiology, Anatomy and Genetics, University of Oxford, Dorothy Crowfoot Hodgkin Building, South Parks Road, Oxford, OX1 3QX UK; 4 Molecular Neurodegeneration Research Group, Nuffield Department of Clinical Neurosciences, University of Oxford, Dorothy Crowfoot Hodgkin Building, South Parks Road, Oxford, OX1 3QU UK; 5 Chinese Academy of Medical Sciences (CAMS), CAMS Oxford Institute (COI), Nuffield Department of Medicine, University of Oxford, Oxford, OX3 7FZ UK; 6 James and Lillian Martin Centre for Stem Cell Research, Sir William Dunn School of Pathology, University of Oxford, South Parks Road, Oxford, OX1 3RE UK

**Keywords:** Induced pluripotent stem cells, Neurodegeneration

## Abstract

Motor neuron diseases such as amyotrophic lateral sclerosis are primarily characterized by motor neuron degeneration with additional involvement of non-neuronal cells, in particular, microglia. In previous work, we have established protocols for the differentiation of iPSC-derived spinal motor neurons and microglia. Here, we combine both cell lineages and establish a novel co-culture of iPSC-derived spinal motor neurons and microglia, which is compatible with motor neuron identity and function. Co-cultured microglia express key identity markers and transcriptomically resemble primary human microglia, have highly dynamic ramifications, are phagocytically competent, release relevant cytokines and respond to stimulation. Further, they express key amyotrophic lateral sclerosis-associated genes and release disease-relevant biomarkers. This novel and authentic human model system facilitates the study of physiological motor neuron-microglia crosstalk and will allow the investigation of non-cell-autonomous phenotypes in motor neuron diseases such as amyotrophic lateral sclerosis.

## Introduction

Motor neuron (MN) diseases such as amyotrophic lateral sclerosis (ALS) are primarily characterized by the degeneration of cortical, brainstem, and spinal cord MNs, resulting in progressive paralysis and premature death. Experimental evidence for ALS supports a pathophysiological model in which a combination of cell-autonomous and non-cell-autonomous factors leads to MN demise^1–3^. In addition to dysregulated MN-intrinsic pathways, e.g., oxidative stress or protein aggregation^4^, there is broad consensus that non-neuronal cells orchestrate a complex neuroinflammatory process in the central nervous system (CNS) of ALS patients^5^. In particular, a wealth of evidence supports a role for microglia, ranging from early *post mortem* and imaging studies^6,7^ to recent biomarker investigations in which microglia-associated inflammatory markers were elevated in the cerebrospinal fluid of ALS samples^8,9^.

Microglia are the resident macrophages of the CNS and execute crucial functions for the maintenance of neuronal homeostasis, for instance, by providing nurture and support to neurons via secretion of soluble factors, clearance of dead cells or misfolded proteins, and protection from infectious agents^10^. Several key ALS-associated genes, notably *SOD1* and *C9ORF72,* are highly expressed in microglia^11,12^. Animal models based on perturbation of various ALS-associated genetic mutations can lead to the development of a pro-inflammatory microglial phenotype; for instance, *C9orf72* knock-out mice show a pro-inflammatory state in myeloid cells and their microglia are toxic to neurons in murine co-cultures^11,13,14^. However, there are distinct species-specific microglial properties that differ between rodents and humans^15^ and many microglial neurodegenerative disease-associated genes do not have appropriate orthologs in mouse^16^. The specific role of microglia in ALS pathophysiology is thus unresolved, and there is a strong need for more authentic disease models using human cells. While multiple iPSC differentiation protocols exist to study phenotypes in ALS patient-derived neurons and astrocytes, in mono- and co-culture^17^, a cell model to study human MN-microglia crosstalk has to our knowledge not previously been reported.

We have previously developed a reproducible protocol for the differentiation of spinal MNs from human induced pluripotent stem cells (iPSCs), providing a viable model to study cell-autonomous disease phenotypes^18,19^. Furthermore, we have established a very efficient differentiation protocol to generate iPSC-derived macrophage precursors^20,21^, which are *MYB*-independent and *RUNX1*- and *PU.1*-dependent, and therefore correspond to primitive, likely yolk sac-derived precursors^22^. During neurodevelopment, yolk sac-derived macrophages (microglia precursors) migrate into the CNS and mature to microglia in concert with neurons through colony stimulating factor 1 receptor (CSF1R) engagement^23–25^. Mimicking embryonic development, we demonstrated that iPSC-derived macrophage precursors adopted microglial properties upon addition of the CSF1R agonist interleukin 34 (IL-34) to the medium and co-culture with iPSC-derived cortical neurons^26^. Co-cultured iPSC-derived microglia expressed relevant microglia markers, displayed dynamic ramifications, and were functionally active with respect to phagocytosis and their expected secretory profile^26^.

Here, we combine both differentiation protocols in a co-culture system of iPSC-derived microglia and iPSC-derived spinal MNs and establish a novel in vitro model to investigate human microglia-MN crosstalk in physiology and disease. MNs in co-culture express MN markers, show neuronal electrophysiological properties in whole-cell patch-clamp electrophysiology and are active in calcium imaging, both spontaneously and after stimulation with potassium chloride. Co-cultured microglia make direct contact with MNs and their neurites, show ramifications with highly dynamic remodeling, have a microglial gene signature similar to primary human microglia, with co-culture supporting their homeostatic state, and also express key ALS-associated genes. Furthermore, they are phagocytically competent and ADP-responsive, release multiple cytokines and chemokines, including the ALS-associated biomarker CHI3L1, and alter their secretory profile and morphology in response to stimulation.

## Results

### MNs differentiated in co-culture medium and in co-culture with microglia retain expression of MN markers

To establish a co-culture of iPSC-derived spinal MNs and microglia, we aimed to mimic embryonic spinal cord development by combining MN and microglia precursors in one dish and then allowing both cell types to mature in concert. In our well-established spinal MN differentiation protocol^18,19,27^, we first promote neural identity using Compound C and Chir99021 and consecutively induce caudalization and ventralization via addition of retinoic acid (RA) and smoothened agonist (SAG). On day in vitro (DIV) 18, MNs are re-plated and final post-mitotic maturation is achieved by additional treatment with Brain-Derived Neurotrophic Factor (BDNF), Glial-Derived Neurotrophic Factor (GDNF), and (2S)-N-[(3,5-Difluorophenyl)acetyl]-L-alanyl-2-phenyl]glycine 1,1-dimethylethyl ester (DAPT). Intending to combine the precursors of both cell types as early as possible without disturbing MN differentiation, we opted to differentiate MNs in monoculture with no changes to the protocol until DIV 21, at which timepoint the neurons already display robust neurite branching. On DIV 21, we aimed to add in the macrophage/microglia precursors, differentiated separately following our highly efficient protocol^20^ (Supp. Fig. 1A), and then mature both cell types in co-culture until at least DIV 35, as 14 days was found to be an optimal co-culture duration in our previous study^26^.

**Figure 1:**
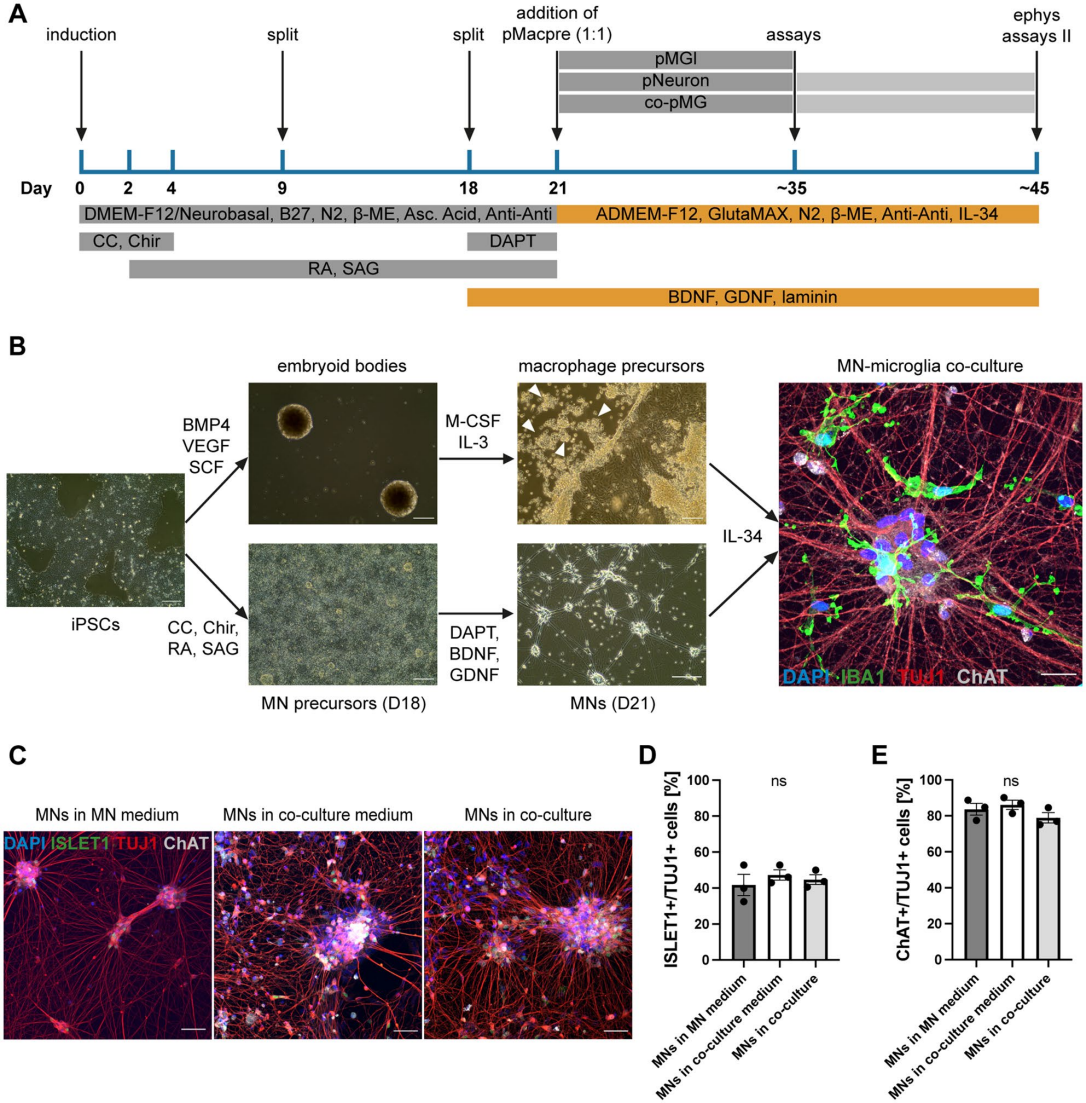
Co-culture of iPSC-derived motor neurons and microglia. (**A**) Overview of protocol for iPSC-derived MN differentiation and co-culture with microglia. On D21, macrophage precursors (pMacpre) were harvested from differentiation factories and replated onto iPSC-derived MNs in a 1:1 ratio. In parallel, microglia (microglia-like, pMGL) and MNs (pNeuron) were differentiated in monocultures. Most assays were conducted after 2 weeks of differentiation (~ D35), while some electrophysiology experiments were additionally performed at ~ D45. (**B**) Left: exemplar images for both differentiation lineages at critical time points of the differentiation. pMacpre are progressively released from embryoid bodies (EBs) into the media (indicated by white arrowheads), while MN precursors are differentiated in a monolayer setup. Right: exemplar image of the MN-microglia co-culture with clear expression of IBA1 in microglia and TUJ1 and ChAT in MNs. Scale bars: 200 μm (left and center images), 25 μm (right image). (**C**) Representative images of MNs in MN medium (left), co-culture medium (center), and co-culture with microglia (right), stained for the MN markers ISLET1 and ChAT. Scale bars: 50 μm. (**D**,**E**) Quantification of the expression of ISLET1 (**D**) and ChAT (**E**) in TUJ1-positive neurons in MN medium, co-culture medium, and co-culture with MNs. Single data points from n = 3 lines from different healthy donors. ns: no significant difference, according to one-way ANOVA and Tukey’s multiple comparisons test.

First, we sought to optimize the culture medium for compatibility with both cell types. We sequentially excluded four immunomodulatory constituents, namely B27 supplement, RA, SAG, and DAPT, from the culture medium on D21, while replacing the DMEM-F12/Neurobasal 50:50 background medium with Advanced DMEM-F12 (ADMEM-F12) plus GlutaMAX and adding in Interleukin-34 (IL-34), to establish optimal culture conditions for microglia differentiation and survival as described previously^26^ (Fig. 1A). Compared with MNs differentiated in normal MN medium, we observed equal expression of the neuronal marker TUJ1 and the MN markers ChAT and ISLET1 in MNs differentiated in co-culture medium on DIV 35 (Supp. Fig. 1B–D). We then combined MN and microglia precursors on DIV 21 and differentiated both cell types together for 14 days (Fig. 1B). The resulting co-cultures showed clear expression of the microglia marker IBA1 and the MN marker ChAT. The expression levels of ChAT and ISLET1 in TUJ1-positive cells were similar for MNs in co-culture and MNs in monoculture differentiated in MN medium and co-culture medium (Fig. 1C–E).

To evaluate whether changes to the medium composition, or co-culture with microglia, affected neuronal survival, we additionally analyzed the expression of the apoptosis marker cleaved caspase 3 (CC3) in TUJ1-positive neuronal cells. We found no significant differences in CC3 expression in TUJ1-positive neurons between the different culture conditions (Supp. Fig. 1E,F).

Together, these results demonstrate that MN differentiation and identity is compatible with microglial co-culture.

### Co-culture with microglia is compatible with the functional neuronal identity of MNs

**Figure 2:**
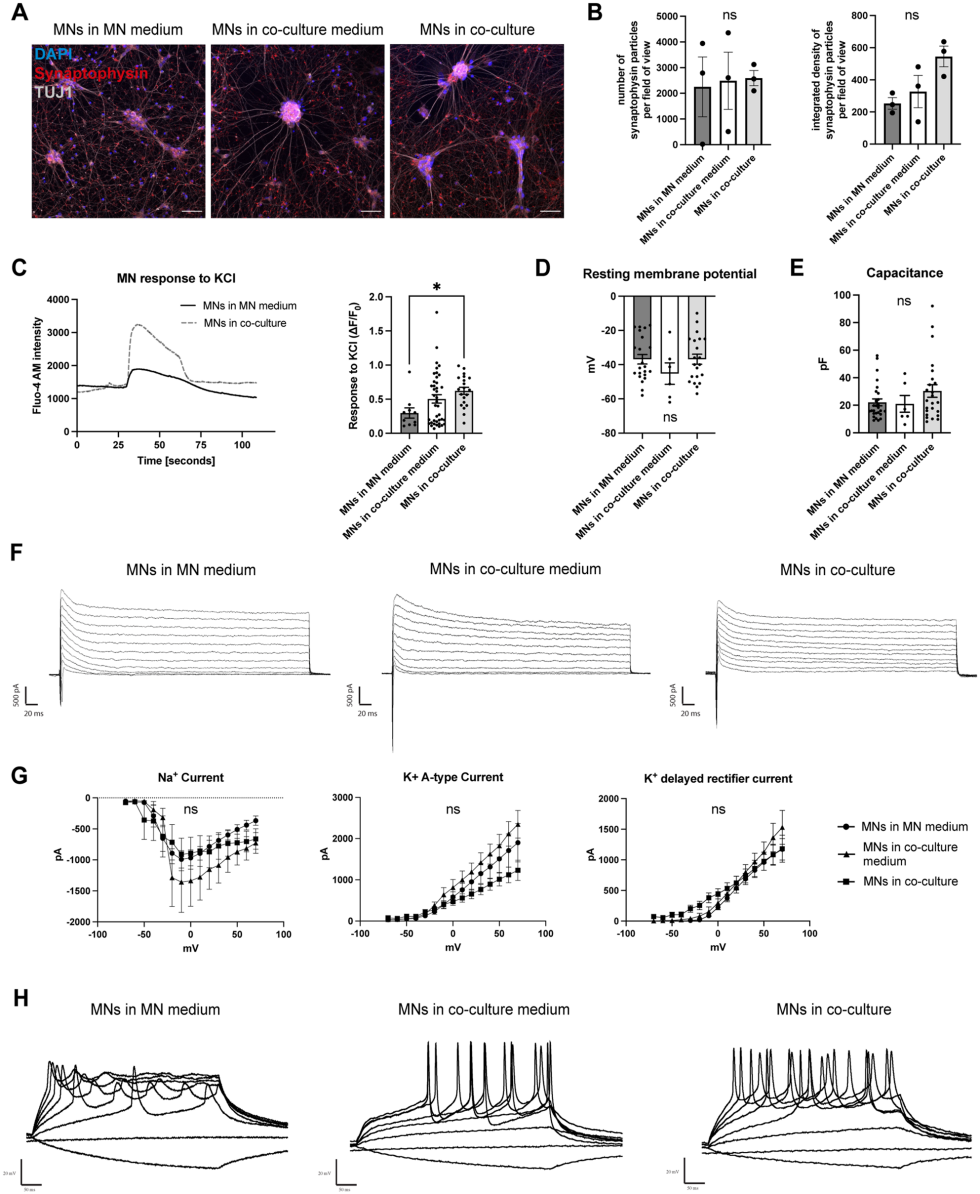
Co-culture with microglia is compatible with MN function. (**A**) Exemplar images of MNs in MN medium, co-culture medium, and co-culture with microglia, stained for the presynaptic marker synaptophysin. Scale bar: 50 μm. (**B**) Quantification of number (left) and integrated density (right) of synaptophysin particles per field of view in MN medium, co-culture medium, and co-culture with microglia. Single data points from n = 3 lines from different healthy donors. (**C**) Left: Analysis of Fluo-4 AM intensity in MNs in monoculture and co-culture shows response to potassium chloride treatment. Right: Quantification of calcium transients after stimulation with potassium chloride. Single data points are individual neurons from n = 1 healthy control line. (**D**,**E**) Quantification of the resting membrane potential (**D**) and cell capacitance (**E**) using whole-cell patch-clamp electrophysiology. Single data points represent individual neurons from n = 3 lines from different healthy donors with n = 3 differentiations each. (**F**) Example images of voltage gated channel currents for MNs in MN medium (left), co-culture medium (center), and co-culture with microglia (right). (**G**) Quantification of the Na_V_ current (left), K_V_ A-type current (center), and K_V_ delayed rectifier current (right) (MNs from n = 3 lines from different healthy donors with n = 3 differentiations each). (**H**) Example images of induced action potentials in MNs in MN medium (left), co-culture medium (center), and co-culture with microglia (right) measured using whole-cell patch-clamp electrophysiology. One-way ANOVA (**B**–**E**) and Two-way repeat measurement ANOVA (**G**) and Tukey’s multiple comparisons test.

We then sought confirmation that MNs retained their functional neuronal identity in co-culture. First, we stained co-cultures and MN monocultures for the pre-synaptic marker synaptophysin and found distinct and localized expression in all conditions (Fig. 2A). Quantification showed no significant differences in synaptophysin expression in co-culture (Fig. 2B). We then performed calcium imaging to analyze calcium oscillations in MNs, measured both spontaneously and after stimulation with potassium chloride. To easily distinguish MNs and microglia in co-culture, we used microglia differentiated from a healthy control line with stable RFP expression^26^. Spontaneous calcium oscillations were observable in co-cultured MNs, while the addition of potassium chloride resulted in typical elevations in intra-cellular calcium (Fig. 2C). Compared with MNs in MN medium, MNs in co-culture medium and co-culture showed an elevated response to potassium chloride (Fig. 2C). Interestingly, in both conditions microglia responded to potassium chloride with the release of calcium into the extracellular space, but this was preceded by calcium uptake in co-cultured microglia (Supp. Fig. 2A), possibly reflecting a direct microglial response to neuronal activation.

In addition, we performed a more extensive functional characterization of MNs in monoculture and co-culture using whole-cell patch-clamp electrophysiology. MNs in MN medium, co-culture medium, and in co-culture with microglia had similar resting membrane potentials and cell capacitances (Fig. 2D,E). Furthermore, we found clear voltage gated channel currents in all conditions (Fig. 2F), with no quantitative differences between MNs in monoculture and co-culture (Fig. 2G). Finally, MNs in all conditions were capable of generating action potentials (APs) after stepwise depolarization (Fig. 2H), with more prominent trains of APs visible in co-culture medium and co-culture.

In summary, these data demonstrate that co-culture with microglia is compatible with MN function.

### Transcriptomic analysis demonstrates a microglial signature

We then sought to analyze the identity of microglia differentiated in monoculture and co-culture with MNs. CD11b was clearly and highly expressed in microglia in co-cultures (Supp. Fig. 3A), rendering it suitable to pull down co-cultured microglia for further transcriptomic analysis, as performed previously^26^. We therefore enriched co-cultured microglia using CD11b-magnetic-activated cell sorting (MACS) from dissociated co-cultures and then subjected them to RNA sequencing analysis, along with their non-terminally differentiated precursors and microglia cultured in monocultures. Microglia in monoculture and co-culture were differentiated in parallel from the same harvest of precursors to avoid transcriptomic differences due to potential batch effects between the different cell types.

First, we compared the identity of our cells with blood monocytes, primary human microglia, and other iPSC-derived microglia grown in monoculture and co-culture. To this end, we obtained and reanalyzed the RNA sequencing dataset from two seminal iPSC microglia publications^28,29^. Comparisons between different datasets have inherent limitations due to differences in sample processing; however, we performed identical mapping, processing, and downstream analyses for all three datasets to optimize the comparability of the sequencing analyses.

First, we compared the expression of *MYB*, *PU.1*, and *RUNX1*, as *bona fide* microglial cells are thought to develop from yolk-sac precursors in an *MYB*-independent and *RUNX1*- and *PU.1*-dependent manner^22^. *MYB* expression was almost absent in our iPSC myeloid cells, similar to primary fetal and adult human microglia (Supp. Fig. 4A). In contrast, *PU.1* and *RUNX1* were highly expressed, providing reassurance that the ontogeny of our cells is in keeping with their assumed primitive hematopoietic origin, in line with our previous observations^22^.

**Figure 3:**
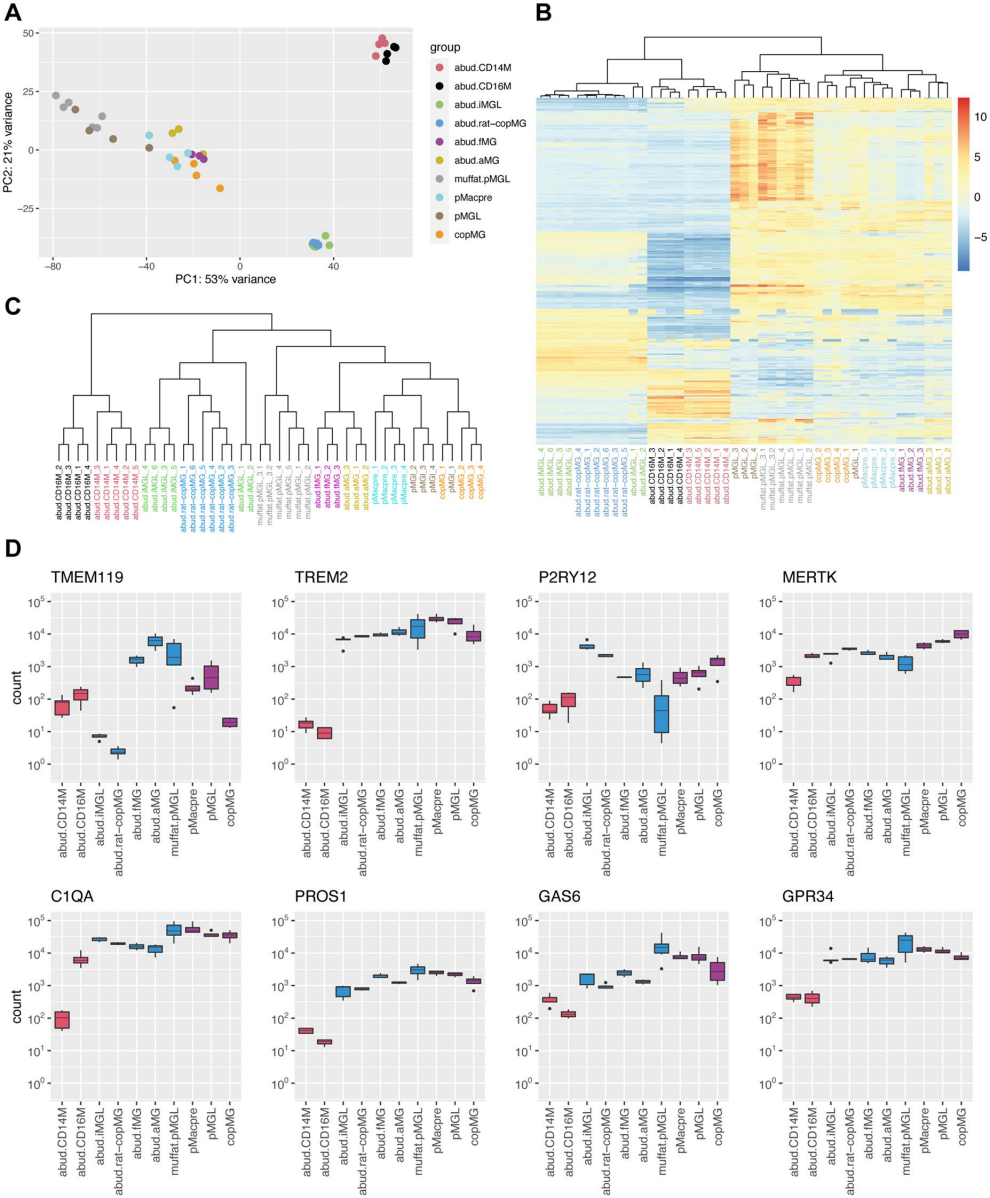
Transcriptomic analysis demonstrates a microglial signature for microglia in monoculture and co-culture. (**A**) Principal component analysis (PCA) plot of VST-transformed RNA sequencing samples based on top 500 most variable genes with biggest variance. Macrophage precursors (pMacpre) and microglia in monoculture (pMGL) and co-culture with MNs (co-pMG) were generated from four different healthy control lines in this study and integrated with samples from Abud et al.^28^ (microglia in monoculture: abud.iMGL, microglia in co-culture with rat neurons: abud.rat-copMG, CD14^+^ blood monocytes: abud.CD14M, CD16^+^ blood monocytes: abud.CD16M, fetal primary human microglia: abud.fMG, adult primary human microglia: abud.aMG) and Muffat et al.^29^ (microglia in monoculture: muffat.pMGL). (**B**) Heatmap of relative VST-transformed values across samples based on top 500 most variable genes with biggest variance with unsupervised hierarchical clustering between samples using the pheatmap 1.0.12 package. (**C**) Dendrogram of unsupervised hierarchical clustering of relative VST-transformed values of samples based on set of ~ 1300 microglial genes identified by Galatro et al.^30^ using the pheatmap 1.0.12 package. (**D**) Box plots of normalized DESeq2 counts for 8 key microglial genes in RNA sequencing (n = 6 samples for abud.iMGL, abud.rat-copMG and muffat.pMGL, n = 5 samples for abud.CD16M, n = 4 samples for abud.CD14M, pMacpre, pMGL and copMG, n = 3 samples for abud.aMG and abud.fMG). Red: blood monocytes from Abud et al.^28^; blue: iPSC microglia and primary human microglia from Abud et al.^28^ and Muffat et al.^29^; purple: microglial samples sequenced in this study.

For exploratory analysis, we performed principal component analysis (PCA) of the top 500 most variable genes with biggest variance. The PCA plot of PC1 versus PC2 broadly showed three groups, with robust reproducibility between the different replicates for each cell type (Fig. 3A). CD14^+^ and CD16^+^ blood monocytes from Abud et al.^28^ grouped together and separated out from a second group containing the iPSC-derived microglia in monoculture and co-culture with rat neurons from Abud et al.^28^ and a more diffuse third group. This group contained the monocultured microglia from Muffat et al.^29^, all of the myeloid/microglial cells generated in this study, and the primary fetal and adult human microglia. Interestingly, on PC1, the monocultured microglia generated in this study were more closely aligned with the microglial monocultures from Muffat et al.^29^, while co-culture with MNs shifted them towards the *bona fide* human microglia.

Unsupervised hierarchical clustering for the top 500 most variable genes with biggest variance broadly confirmed these observations (Fig. 3B). The microglia cultured in monoculture and co-culture with rat neurons from Abud et al.^28^ clustered together with the blood monocytes, possibly reflecting their hematopoietic origin in line with the differentiation protocol^28^. The precursors and co-cultured microglia from this study clustered with the fetal and adult microglia, while 3 of the 4 microglial monoculture samples clustered with the monocultured microglia from Muffat et al.^29^. We additionally performed unsupervised hierarchical clustering based on the expression of a set of ~ 1300 microglial key genes as identified by Galatro et al.^30^ (Fig. 3C, Supp. Fig. 4B). Here, the blood monocytes formed a separate cluster from all other cell types. Intriguingly, the myeloid cells generated in this study clustered closely with the *bona fide* primary human microglia.

Finally, we compared our microglia grown in monoculture and co-culture and identified 1569 differentially expressed genes (Supp. Fig. 5A). The genes upregulated in co-culture showed enrichment of the GO term “regulation of neurotransmitter levels” and three cytokine-associated terms (Supp. Fig. 5B). Some of the genes associated with these terms such as *SYT4* (Supp. Fig. 5C) have high expression in neurons and could be differentially expressed due to low-level neuronal contamination of the CD11b-MAC-sorted co-culture microglial population or possible phagocytic engulfment of neuronal material by microglia. However, homeostatic microglia-specific genes including *LAG3* and *IRF4* also showed up-regulation in co-culture (Supp. Fig. 5C). The genes downregulated in co-culture had the highest enrichment score for extracellular matrix-associated GO terms (Supp. Fig. 5D), with downregulation of several collagen- and cell-to-matrix-associated genes, including *COL4A1*, *FN1* and *FBN1*, and matrix metalloproteinases such as *MMP16* (Supp. Fig. 5E).

To assess the difference between microglia in monoculture and co-culture morphologically, we additionally analyzed the microglial ramifications (Supp. Fig. 6A–D). Compared with microglia in monoculture, microglia co-cultured with MNs showed similar branch length but an increased number of branch points and endpoints, reflecting an increased ramified state in co-culture and corroborating that co-culture supports a homeostatic microglial identity. Finally, quantification of cleaved caspase 3 in IBA1-positive microglia showed minimal expression in monoculture and co-culture, reflecting a healthy microglial state in both culture conditions (Supp. Fig. 6E).

Together, these data demonstrate the microglial identity of our microglial cells. In particular, co-cultured microglia showed close resemblance with *bona fide* primary human microglia.

### Microglia express signature microglial and ALS-associated genes

We then sought to compare the expression of key myeloid/microglial markers associated specifically with microglia but not with blood monocytes as defined by previous studies^31–33^ in the different myeloid cells in our RNA-seq analysis. We found clear expression and enrichment of *P2RY12*, *MERTK*, *TREM2*, *TMEM119*, *PROS1*, *C1QA*, *GAS6*, and *GPR34* (Fig. 3D, Supp. Fig. 7A). Compared with the classical blood monocytes (abud.CD14M), *TREM2*, *MERTK*, *C1QA*, *PROS1*, *GAS6*, and *GPR34* were particularly and consistently enriched across the different iPSC-derived and primary microglial cells.

In addition, we investigated the expression of key ALS-associated genes across the different myeloid samples (Supp. Fig. 7B)*.* We found high expression of *C9ORF72*, *SOD1*, *TARDBP*, and *FUS*. The levels of *C9ORF72* were slightly higher in blood monocytes than in the different microglial cells, and its expression increased in co-culture. Intriguingly, the expression of the putative ALS biomarkers *CHIT1*, *CHI3L1*, and *MCP-1* was strongly enriched in most microglial cells compared with blood monocytes, suggesting that microglia are likely to be a key source of these biomarkers and a suitable model to study their release in vitro.

**Figure 4:**
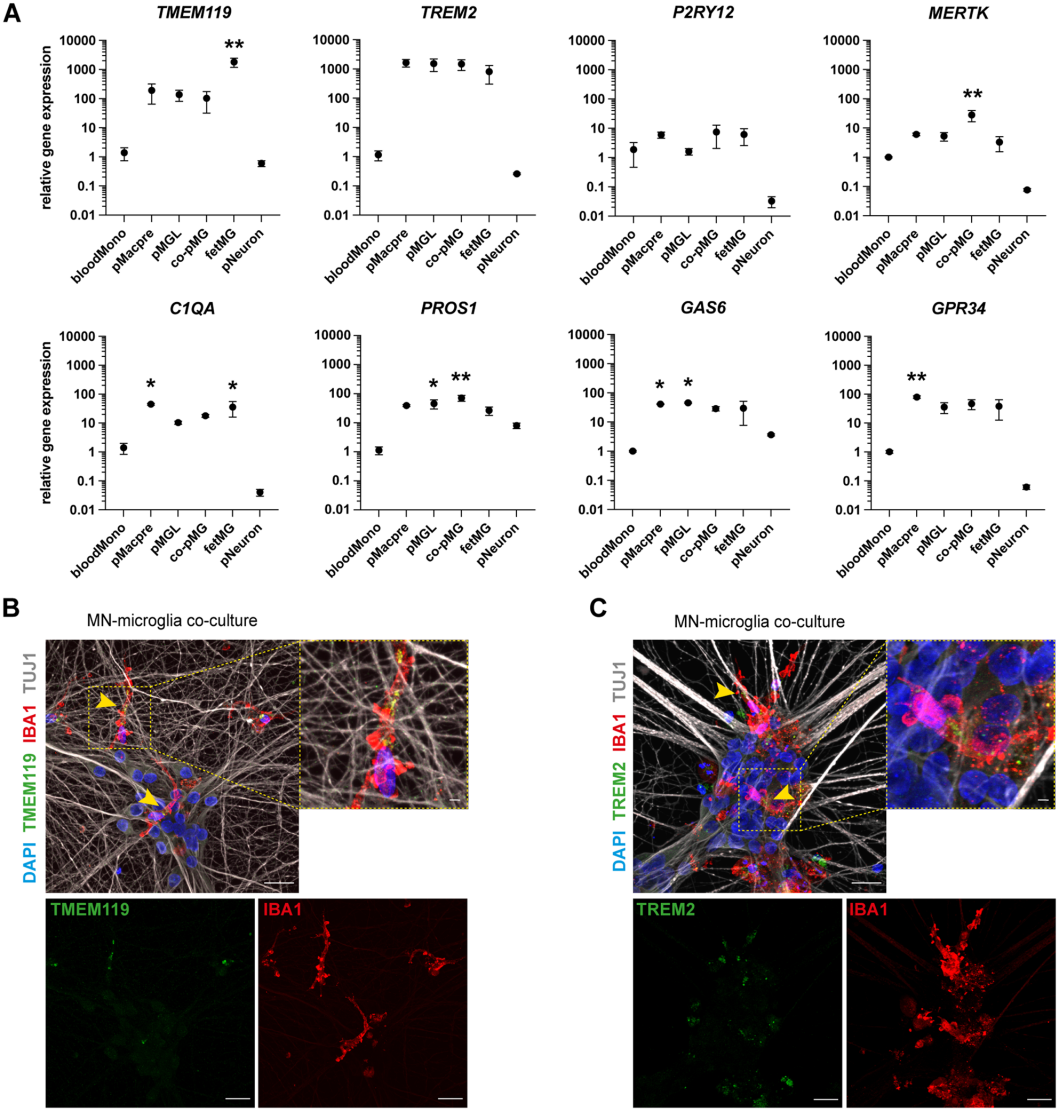
iPSC-derived microglia express macrophage-/microglia markers at gene and protein level. (**A**) Quantification of the relative gene expression of multiple macrophage/microglia markers by RT-qPCR in iPSC-derived macrophage precursors (pMacpre), microglia in monoculture (pMGL), microglia from co-culture after CD11b-MACS (co-pMG), motor neurons in monoculture differentiated in co-culture medium (pNeuron) (all n = 3 lines from different healthy donors), human fetal microglia (fetMG, n = 3 donors), and human blood monocytes (bloodMono, n = 3 healthy donors). Relative expression was calculated using the 2^-ΔΔCt^ method, with *GAPDH* as an endogenous control and normalization to bloodMono. One-way ANOVA and Dunnett’s multiple comparisons test versus bloodMono. (**B**,**C**) Representative images of microglia in co-culture with MNs. Yellow arrowheads and close-ups indicate co-expression of the macrophage/microglia markers TMEM119 (**B**) and TREM2 (**C**) in IBA1-positive microglia. Scale bars (including insets): 25 μm.

To validate the expression of the key microglial markers, we performed RT-qPCR analysis of a different sample set including CD14^+^ blood monocytes, fetal primary human microglia, precursors, microglial monocultures, CD11b-MAC-sorted co-cultured microglia, and, additionally, monocultured MNs differentiated in co-culture medium as a negative control (Fig. 4A). We confirmed MN identity by RT-qPCR for *ChAT*, which showed strong enrichment in MNs and moderate expression in the MAC-sorted co-culture microglia, likely as a result of low neuronal contamination of the sorted microglial population (Supp. Fig. 7C). In general, we found substantial overlap between our RNA-sequencing analysis and qPCR data. Notably, most myeloid/microglial genes were strongly enriched in all myeloid cells compared with MNs, with the notable exception of *PROS1* and *GAS6* (Fig. 4A). Particularly, *TMEM119* and *TREM2* showed high enrichment in the microglial cells compared with blood monocytes. Furthermore, most genes showed similar expression in the iPSC-derived microglial cells and fetal human microglia, with the exception of *TMEM119*, which, in line with our RNA-seq results (Fig. 3D), was most highly expressed in the fetal microglia (Fig. 4A). Finally, we stained co-cultures for TMEM119 and TREM2 and confirmed clear, localized, and high expression of both markers within co-cultured microglia (Fig. 4B,C; Supp. Fig. 7D).

In summary, these data confirm the microglial identity of our iPSC-derived microglial cells and their utility as a model system to study key ALS-associated genes.

### Microglia show highly dynamic ramifications

**Figure 5:**
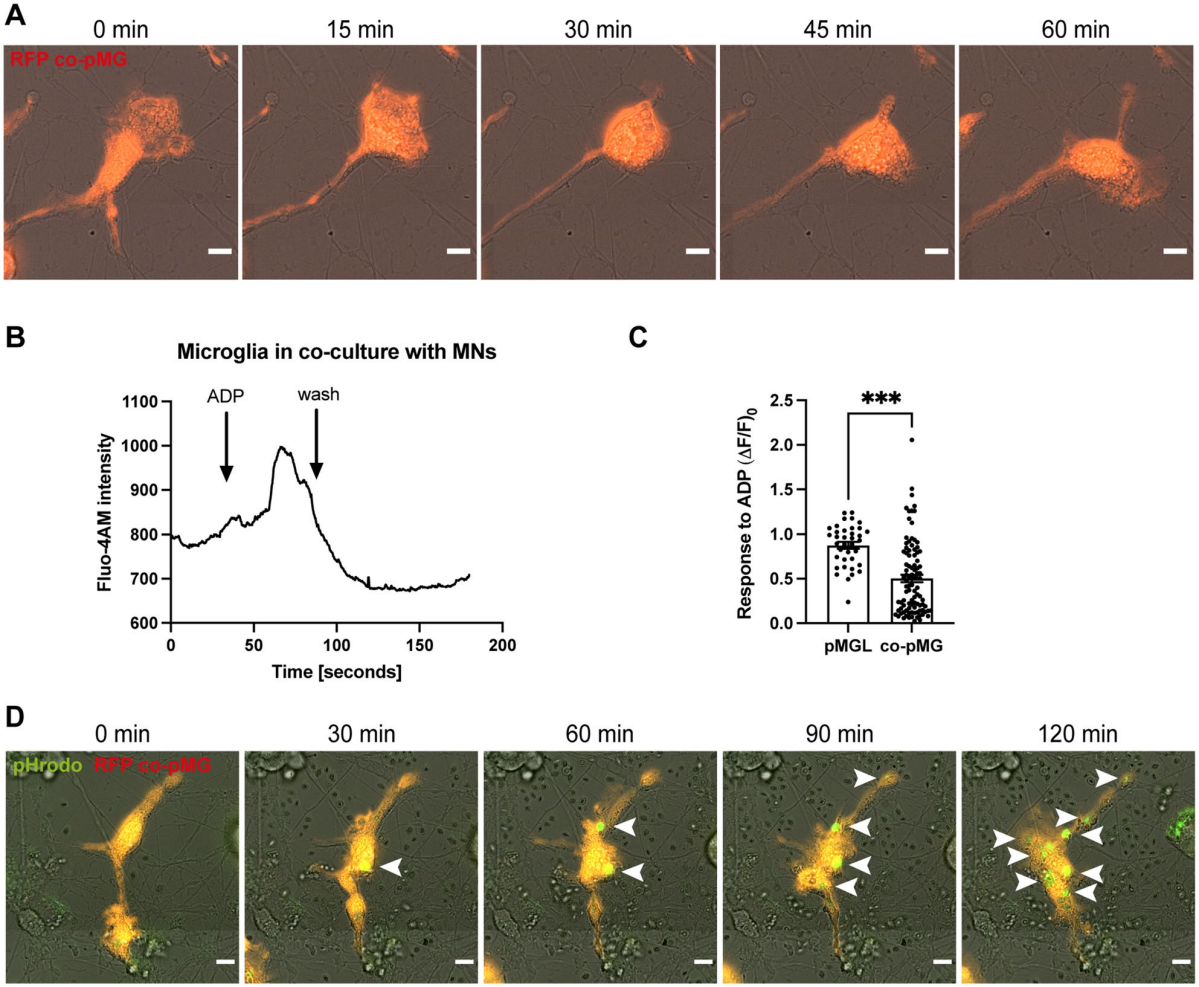
Microglia are highly motile, ADP-reponsive, and phagocytically competent. (**A**) Live imaging of RFP-expressing microglia in co-culture (co-pMG) over 60 min shows highly dynamic remodeling of microglial ramifications. Scale bar: 10 μm. (**B**) Analysis of Fluo-4 AM intensity in co-pMG shows response to ADP treatment. (**C**) Quantification of calcium transients in microglia in monoculture (pMGL) and co-pMG using the fluorescent probe Fluo-4 AM after stimulation with ADP. Single data points are individual neurons from one healthy control line. Two-tailed unpaired t test. (**D**) Live imaging of RFP-expressing co-pMG over 120 min after addition of pHrodo green zymosan bioparticles shows progressive phagocytic uptake. White arrowheads: phagocytosed particles. Scale bar: 10 μm.

Having demonstrated the microglial signature of our iPSC-derived microglia, we then investigated their functional properties. *Bona fide* microglia are continuously active and constantly scan the surrounding environment using their highly ramified processes. Therefore, we analyzed the motility of co-cultured microglia by live imaging using microglia differentiated from an iPSC line with stable expression of RFP, which allows for easy identification and distinction from neurons in co-culture. Live-imaging with ~ 2 images/min over one hour revealed highly ramified cells with very dynamic remodeling of their ramifications (Movie S1, Fig. 5A). Co-cultured microglia were constantly active and interacted closely with neurons and their neurites, mostly maintaining their territories.

### Microglia respond to ADP stimulation

iPSC microglia but not blood monocyte-derived macrophages have previously been shown to respond to ADP stimulation^34^. We therefore stimulated microglia in monoculture and co-culture with ADP and analyzed their response using calcium imaging. Both microglia in monoculture and co-culture were responsive to ADP stimulation (Fig. 5B,C). Interestingly, co-cultured microglia showed a significantly reduced response to ADP, possibly reflecting their homeostatic state in co-culture.

### Microglia are phagocytically competent

We then evaluated the phagocytic competence of microglia in co-culture, using pHrodo-labelled yeast particles that fluoresce after phagocytic uptake due to progressive lysosomal acidification. Live imaging over 2 h upon addition of these particles revealed increasing visibility of fluorescent dots in co-cultured microglia (Movie S2, Fig. 5D), demonstrating that microglia are phagocytically competent in co-culture.

### Microglia respond to pro-inflammatory stimulation with morphological changes and clustering behavior

**Figure 6:**
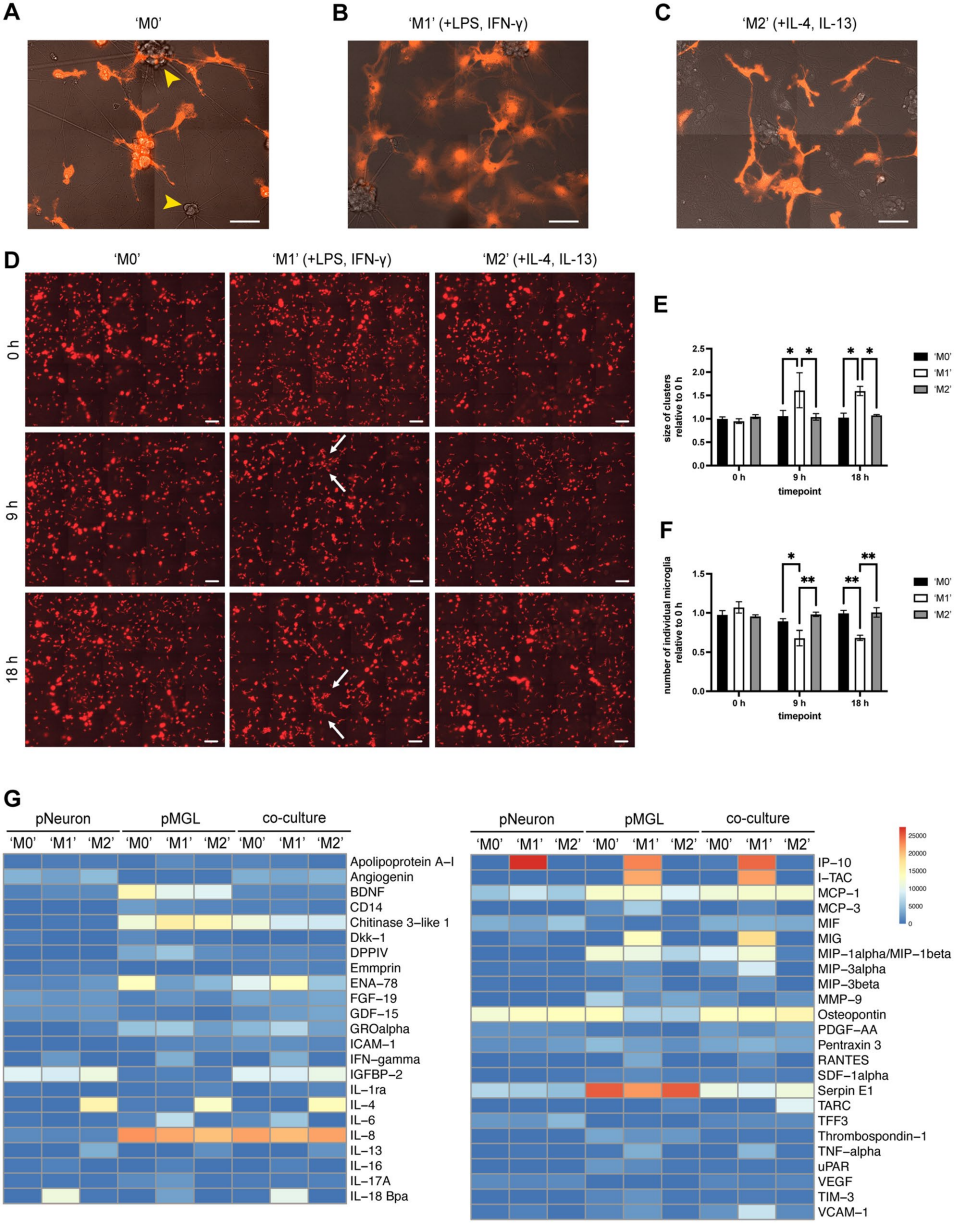
Microglia in co-culture respond to stimulation with morphological changes and clustering behavior and release cytokines in naïve and stimulated conditions. (**A**–**C**) Exemplar live-imaging pictures of RFP microglia in co-culture with MNs. Cells were either treated with vehicle (‘M0’, **A**), LPS/IFN-γ to induce a pro-inflammatory phenotype (‘M1’, **B**) or IL-4/IL-13 to induce an anti-inflammatory phenotype (‘M2’, **C**). Yellow arrowheads indicate MN clusters in co-culture. Scale bar: 50 μm. (**D**) Representative live-imaging images of RFP microglia in co-culture before stimulation (0 h), and after 9 h and 18 h of treatment with LPS/IFN-γ or IL-4/IL-13. White arrows indicate examples of microglial clusters. Scale bar: 250 μm. (**E**) Quantification of the size of microglial clusters in co-culture at different time points after stimulation relative to 0 h-time point (n = 3 lines from different healthy donors). (**F**) Quantification of the number of individual microglial cells in co-culture at different time points after stimulation relative to 0 h-time point (all n = 3 lines from different healthy donors). Two-way repeat measurement ANOVA and Tukey’s multiple comparisons test. (**G**) Cytokine release into the culture supernatant from pNeuron, pMGL, and co-cultures was measured using the Proteome Profiler Human XL Cytokine Array Kit. Cells were either treated with vehicle (‘M0’), LPS/IFN-γ (‘M1’) or IL-4/IL-13 (‘M2’) for 18 h. Pooled supernatant from n = 3 lines from different healthy donors was used for each condition. Data represent the mean of two technical replicates per condition and are presented as a heatmap of arbitrary units (a.u.), where blue indicates low expression and red reflects high release. Only cytokines with a minimum expression of 1000 a.u. in at least one condition are shown. For the full list, see Supp. Table 1.

We then sought to evaluate if microglia in co-culture respond to pro-inflammatory and anti-inflammatory stimulation. First, we assessed the morphological response to stimulation with Lipopolysaccharide (LPS)/Interferon-γ (IFN-γ) or IL-4/IL-13 to induce contrasting phenotypes (simplistically termed ‘M1’ or ‘M2’, respectively, versus ‘M0’). Live-imaging after 18 h of stimulation revealed amoeboid morphology and clustering of co-cultured RFP-expressing microglia in pro-inflammatory (LPS/IFN-γ) conditions, while no overt changes were observable after induction with IL-4/IL-13 (Fig. 6A–C). To study changes in clustering more closely, we also performed live-imaging of co-cultures before treatment and 9 h and 18 h after the addition of LPS/IFN-γ or IL-4/IL-13 (Fig. 6D). Quantification of the number of individual microglial cells and the size of microglial clusters showed no differences before stimulation (Fig. 6E,F). However, treatment with LPS/IFN-γ led to a significant increase in cluster size after both 9 h and 18 h of stimulation, compared with unstimulated or IL-4/IL-13 conditions (Fig. 6E). In keeping with this result, the number of individual microglial cells was significantly reduced after 9 h and 18 h of treatment with LPS/IFN-γ (Fig. 6F).

In summary, these findings show that microglia in co-culture with MNs actively respond to stimulation with morphological changes and clustering behavior, demonstrating the dynamic behavior expected of microglial cells.

### Microglia release pro-inflammatory and anti-inflammatory cytokines

Finally, we evaluated the microglial release of cytokines and chemokines in monoculture and co-culture. We used a membrane-based supernatant proteome array, which allows for the detection of 105 different cytokines and chemokines. We analyzed microglial monocultures, MNs, and co-cultures, all differentiated in the same co-culture medium with/without LPS/IFN-γ or IL-4/IL-13. Unstimulated MNs released only a small number of cytokines, including IGFBP-2 or osteopontin, while stimulation of MNs with LPS/IFN-γ upregulated the release of a few additional cytokines such as IP-10 (Fig. 6G, Supp. Table 1). Treatment of MNs with IL-4/IL-13 did not result in significant changes. In contrast, microglial monocultures and co-cultures secreted many more cytokines, both in naïve and stimulated conditions. Treatment with LPS/IFN-γ led to the release of several key inflammatory mediators, for instance, IL6, TNF, RANTES/CCL5 and MIG/CXCL9, in microglial monocultures and co-cultures (Fig. 6G, Supp. Table 1, Supp. Fig. 8A). In comparison with microglial monocultures, co-cultures showed a moderately attenuated secretion profile, for instance with apparent downregulation of CHI3L1 or serpin E1. In keeping with these data, we detected substantial release of serpin E1, a serine protease inhibitor, from monocultured microglia by ELISA, which was abolished in co-culture (Supp. Fig. 8B), and *serpin E1* was also downregulated in co-cultured microglia compared with monocultures in our RNA-seq analysis (Supp. Fig. 5E). Similarly, ELISA validation for CHI3L1, a potential biomarker in the CSF of ALS patients thus far associated with astrocytes^5,8^, showed no release of CHI3L1 from MNs but a substantial secretion from microglia in monoculture, which was at lower levels in co-culture supernatants (Supp. Fig. 8C).

Together, microglia released multiple cytokines in both unstimulated and stimulated conditions, with a moderately altered secretion profile in co-culture.

## Discussion

We have established a reliable co-culture of iPSC-derived microglia and MNs, allowing the study of MN-microglia crosstalk in an authentic human model system, with particular relevance for ALS research. MNs in co-culture express key markers and have the expected neuronal electrophysiological properties. Co-cultured microglia fulfill multiple established criteria for microglial cell identity – they transcriptomically resemble *bona fide* primary human microglia, express key microglial markers, have highly dynamic ramifications, are phagocytically competent, and release a battery of cytokines, both in naïve conditions and upon stimulation. Of particular interest for ALS research, co-cultured microglia express key ALS-associated genes and release disease-relevant biomarkers. Importantly, we show that microglial monocultures also represent a valid microglia model, allowing the study of cell-autonomous phenotypes in a simplified system, while co-culture supports a homeostatic microglial state and is best suited for the investigation of non-cell-autonomous effects.

Various methods for the differentiation of iPSC-derived microglia have been published in recent years, and there is great overlap between the functional properties of these cells^17^. However, thus far, only our protocol has been shown to produce *MYB*-independent and *PU.1*-and *RUNX1*-dependent primitive macrophages through gene knock-out^22^. We corroborate this finding here by RNA sequencing, showing that *MYB* expression is almost absent in our iPSC-derived myeloid cells and thereby similar to primary human microglial cells, while *PU.1* and *RUNX1* are highly expressed. While comparing sequencing datasets poses challenges due to differences in sample processing, we integrated our RNA-sequencing data with the datasets from two seminal protocols for the differentiation of microglial cells^28,29^ and limited batch effects due to simultaneous bioinformatical processing of the raw data. Direct comparison indicates that our monocultured microglia transcriptomically resemble the microglia from established protocols^29^, while co-cultured microglia are close to *bona fida* primary human microglia^28^. Importantly, our differentiation protocol is relatively simple and highly efficient, allowing for easy scaling up of production for large-scale and drug screening experiments^26^.

The combined findings from our earlier study^26^ and this study show that microglial monocultures are a viable, simple and reliable model to study microglial function as well as cell-autonomous phenotypes, with great relevance for neurodegenerative disease research. In this study, we focused on establishing the co-culture of microglia and spinal MNs. Many other microglia differentiation protocols have also shown compatibility with neurons, either through direct co-culture or by transplantation into animals^17^, but we provide the first human model system to investigate microglia-spinal MN crosstalk. As would be expected, and in keeping with other protocols, we also demonstrate that co-culture supports a homeostatic microglial state, with increased ramifications, higher expression of some homeostatic genes, reduced ADP response, and a moderately attenuated secretion profile. Interestingly, we found reduced serpin E1 in co-culture supernatants compared to iPSC microglia monoculture supernatants in this study, whereas serpin E1 secretion was upregulated in our previous co-culture of cortical neurons and microglia^26^. This result could indicate that microglia in co-culture specifically respond to the neuronal subtype they are cultured with, a phenomenon which should be further explored in future studies.

Importantly, we show that several key ALS-associated genes, notably *C9ORF72,* are expressed in microglia in monoculture and co-culture, rendering them a suitable model to study cell-autonomous and non-cell-autonomous phenotypes using patient-derived cells. Furthermore, we found several candidate ALS biomarkers to be highly expressed in the microglial cells, including *CHIT1* and *CHI3L1*, and confirmed CHI3L1 release from microglia in monoculture and co-culture through a supernatant array and ELISA. As CHI3L1 has previously been hypothesized to be primarily released by astrocytes^5,8^, future studies should therefore also evaluate microglia as a potential source for increased CHI3L1 secretion.

There is an urgent need to better understand the role of microglia in neurodegenerative diseases such as ALS. We offer a new tool that we envisage will further our understanding of MN-microglial crosstalk and the microglial component of ALS.

## Materials and methods

### Ethics statement

All human material (blood RNA, primary microglia RNA, iPSCs) used in this study was derived after signed informed consent: for blood, according to University of Oxford OHS policy document 1/03; all procedures related to the use of the primary microglia followed established institutional (McGill University, Montreal, QC, Canada) and Canadian Institutes of Health Research guidelines for the use of human cells; for iPSC, with approval from the South Central Berkshire Research Ethics Committee, U.K. (REC 10/H0505/71). The blood RNA and primary microglia RNA samples have been published previously^26^, as have the iPSC lines (see below).

### Generation and culture of iPSC lines

Four healthy control iPSC lines, SFC840-03-03 (female, 67 years old,^35^), SFC841-03-01 (male, 36,^18^), SFC856-03-04 (female, 78,^36^), OX3-06 (male, 49,^37^), generated from skin biopsy fibroblasts and characterized as described before, were used in this study. Additionally, the previously reported^26^ line AH016-3 Lenti_IP_RFP (male, 80 years old), which constitutively expresses Red Fluorescent Protein (RFP) under continuous puromycin selection, was used for some live-imaging experiments.

iPSCs were cultured in mTeSR™1 (StemCell Technologies) or OXE8 medium^38^ on Geltrex™ (Thermo Fisher)-coated tissue culture plates with daily medium changes. Passaging was done as clumps using EDTA in PBS (0.5 mM). Cells were initially expanded at low passage to create a master stock, which was used for all experiments to ensure consistency. Cells were regularly tested negative for mycoplasma using MycoAlert™ Mycoplasma Detection Kit (Lonza).

### Motor neuron differentiation

iPSCs were differentiated to MNs according to our previously published protocol^18,19,27^. Briefly, neural induction of iPSC monolayers was performed using DMEM-F12/Neurobasal 50:50 medium supplemented with N2 (1X), B27 (1X), 2-Mercaptoethanol (1X), Antibiotic–Antimycotic (1X, all ThermoFisher), Ascorbic Acid (0.5 μM), Compound C (1 μM, both Merck), and Chir99021 (3 μM, R&D Systems). After two days in culture, Retinoic Acid (RA, 1 μM, Merck) and Smoothened Agonist (SAG, 500 nM, R&D Systems) were additionally added to the medium. Two days later, Compound C and Chir99021 were removed from the medium. After another 5 days in culture, neural precursors were dissociated using accutase (ThermoFisher), and split 1:3 onto Geltrex™-coated tissue culture plates in medium supplemented with Y-27632 dihydrochloride (10 μM, R&D Systems). After one day, Y-27632 dihydrochloride was removed from the medium, and then the cells were cultured for another 8 days with medium changes every other day. For terminal maturation, the cells were dissociated on day in vitro (DIV) 18 using accutase and plated onto coverslips or tissue culture plates coated with polyethylenimine (PEI, 0.07%, Merck) and Geltrex™ or tissue culture dishes coated with PDL (Sigma-Aldrich)/ Laminin (R&D Systems)/ Fibronectin (Corning). For this step, the medium was additionally supplemented with BDNF (10 ng/mL), GDNF (10 ng/mL), Laminin (0.5 μg/mL, all ThermoFisher), Y-27632 dihydrochloride (10 μM), and DAPT (10 μM, R&D Systems). Three days later, Y-27632 dihydrochloride was removed from the medium. After another three days, DAPT was removed from the medium. Full medium changes were then performed every three days.

For MNs differentiated in co-culture medium alone, all steps were performed similarly until three days after the terminal re-plating (D21). MNs were then cultured in co-culture medium as described below.

### Differentiation to macrophage precursors

iPSCs were differentiated to macrophage/microglia precursors as described previously^20,21^. Briefly, embryoid body (EB) formation was induced by seeding iPSCs into Aggrewell 800 wells (STEMCELL Technologies) in OXE8^38^ or mTeSR™1 medium supplemented with Bone Morphogenetic Protein 4 (BMP4, 50 ng/mL), Vascular Endothelial Growth Factor (VEGF, 50 ng/mL, both Peprotech), and Stem Cell Factor (SCF, 20 ng/mL, Miltenyi Biotec). After four days with daily medium changes, EBs were transferred to T175 flasks (~ 150 EBs each) and differentiated in X-VIVO15 (Lonza), supplemented with Interleukin-3 (IL-3, 25 ng/mL, R&D Systems), Macrophage Colony-Stimulating Factor (M-CSF, 100 ng/mL), GlutaMAX (1X, both ThermoFisher), and 2-Mercaptoethanol (1X). Fresh medium was added weekly. After approximately one month, precursors emerged into the supernatant and could be harvested weekly. Harvested cells were passed through a cell strainer (40 μM, Falcon) and either lysed directly for RNA extraction or differentiated to microglia in monoculture or co-culture as described below.

### Motor neuron-microglia co-culture

Three days after the final re-plating of differentiating MNs (DIV21), macrophage/microglia precursors were harvested as described above and resuspended in co-culture medium comprised of Advanced DMEM-F12 (ThermoFisher) supplemented with GlutaMAX (1X), N2 (1X), Antibiotic–Antimycotic (1X), 2-Mercaptoethanol (1X), Interleukin-34 (IL-34, 100 ng/mL, Peprotech), BDNF (10 ng/mL), GDNF (10 ng/mL), and Laminin (0.5 μg/mL). MNs were quickly rinsed with PBS, and macrophage/microglia precursors re-suspended in co-culture medium were added to each well. Co-cultures were then maintained for at least 14 days before assays were conducted as described below. Half medium changes were performed every 2–3 days.

For comparisons between co-cultures and monocultures, MNs and monocultured microglia were also differentiated alone in co-culture medium.

### Immunofluorescence

Cells cultured on coverslips were pre-fixed with 2% paraformaldehyde in PBS for 2 min and then fixed with 4% paraformaldehyde in PBS for 15 min at room temperature (RT). After permeabilization and blocking with 5% donkey/goat serum and 0.2% Triton X-100 in PBS for 1 h at RT, the coverslips were incubated with primary antibodies diluted in 1% donkey/goat serum and 0.1% Triton X-100 in PBS at 4 °C ON. The following primary antibodies were used: rabbit anti-cleaved caspase 3 (1:400, 9661S, Cell Signaling), mouse anti-ISLET1 (1:50, 40.2D6, Developmental Studies Hybridoma Bank), mouse anti-TUJ1 (1:500, 801201, BioLegend), rabbit anti-TUJ1 (1:500, 802001, BioLegend), chicken anti-TUJ1 (1:500, GTX85469, GeneTex), rabbit anti-IBA1 (1:500, 019-19741, FUJIFILM Wako Pure Chemical Corporation), goat anti-IBA1 (1:500, ab5076, abcam), rabbit anti-synaptophysin (1:200, ab14692, abcam), goat anti-ChAT (1:100, ab114P, abcam), rat anti-TREM2 (1:100, MAB17291-100, R&D Systems), rabbit anti-TMEM119 (1:100, ab185337, abcam), rat anti-CD11b (1:100, 101202, BioLegend).

After three washes with PBS-0.1% Triton X-100 for 5 min each, coverslips were incubated with corresponding fluorescent secondary antibodies Alexa Fluor^®^ 488/568/647 donkey anti-mouse/rabbit/rat/goat, goat anti-chicken (all 1:1000, all ThermoFisher). Coverslips were then washed twice with PBS-0.1% Triton X-100 for 5 min each and incubated with 4′,6-diamidino-2-phenylindole (DAPI, 1 µg/mL, Sigma-Aldrich) in PBS for 10 min. After an additional 5 min-washing step with PBS-0.1% Triton X-100, the coverslips were mounted onto microscopy slides using ProLong™ Diamond Antifade Mountant (ThermoFisher). Confocal microscopy was then performed using an LSM 710 microscope (Zeiss).

### Quantification of MN differentiation efficiency

For the analysis of neuronal and MN markers after differentiation, three z-stacks (2 µm intervals) of randomly selected visual fields (425.1 × 425.1 µm) were taken for each coverslip at 20 × magnification. The ratios of TUJ1-positive, ChAT-positive, ISLET1-positive, ChAT-positive/ TUJ1-positive, and ISLET1-positive/ TUJ1-positive cells were then quantified using Fiji in a blinded fashion.

### Quantification of microglial marker expression

For the analysis of microglial markers in monoculture and co-culture, three z-stacks (1 µm intervals) of randomly selected visual fields (212.55 × 212.55 µm) were taken for each coverslip at 40 × magnification. The expression of CD11b, TMEM119, and TREM2 in IBA1-positive cells in monoculture and co-culture was then quantified using Fiji.

### Quantification of apoptosis

For the analysis of apoptosis in neurons, five z-stacks images of randomly selected visual fields (212.55 × 212.55 µm) were taken at 40 × magnification for each coverslip. The ratios of cleaved caspase 3/ TUJ1-positive cells were then quantified for neurons in monoculture and co-culture in a blinded fashion. For the analysis of apoptosis in microglia, three z-stacks images of randomly selected visual fields (212.55 × 212.55 µm) were taken at 40 × magnification for each coverslip. The ratios of cleaved caspase 3/ IBA1-positive cells were then quantified for microglia in monoculture and co-culture.

### Analysis of microglial ramifications

For the analysis of microglial ramifications, five z-stacks images of randomly selected visual fields (212.55 × 212.55 µm) were taken at 40 × magnification for each coverslip. To analyze the branching of IBA1-positive microglia in monoculture and co-culture, the average branch length, number of branch points and number of branch endpoints was determined using 3DMorph^39^, a Matlab-based script for the automated analysis of microglial morphology.

### RNA isolation

From the same harvest, macrophage precursors (pMacpre) were either lysed directly or differentiated to microglia in monoculture (pMGL) or microglia in co-culture with MNs (co-pMG) for 14 days. pMGL were rinsed with PBS and directly lysed in the dish. For both pMacpre and pMGL, RNA was extracted using an RNAeasy Mini Plus kit (Qiagen) according to the manufacturer’s instructions. Co-cultures were first dissociated by 15 min incubation with papain (P4762, Sigma-Aldrich) diluted in accutase (20 U/mL) and gentle trituration based on a previously published protocol^40^. The cell suspension was then passed through a cell strainer (70 μm, Falcon) to remove cell clumps. To extract co-pMG, magnetic-activated cell sorting (MACS) was then performed using CD11b-MACS beads (130–093-634, Miltenyi Biotec) according to the manufacturer’s instructions. The panned cell population was lysed for RNA extraction using an RNAeasy Micro kit (Qiagen) according to the manufacturer’s instructions. In addition, RNA from human fetal microglia and blood monocytes from three different healthy genetic backgrounds was re-used from our previous study^26^.

### RNA-sequencing

RNA from the four different healthy control lines (listed earlier) per condition (pMacpre, pMGL, co-pMG) was used for RNA sequencing analysis. Material was quantified using RiboGreen (Invitrogen) on the FLUOstar OPTIMA plate reader (BMG Labtech) and the size profile and integrity analysed on the 2200 or 4200 TapeStation (Agilent, RNA ScreenTape). RIN estimates for all samples were between 9.2 and 9.9. Input material was normalised to 100 ng prior to library preparation. Polyadenylated transcript enrichment and strand specific library preparation was completed using NEBNext Ultra II mRNA kit (NEB) following manufacturer’s instructions. Libraries were amplified (14 cycles) on a Tetrad (Bio-Rad) using in-house unique dual indexing primers (based on^41^). Individual libraries were normalised using Qubit, and the size profile was analysed on the 2200 or 4200 TapeStation. Individual libraries were normalised and pooled together accordingly. The pooled library was diluted to ~ 10 nM for storage. The 10 nM library was denatured and further diluted prior to loading on the sequencer. Paired end sequencing was performed using a NovaSeq6000 platform (Illumina, NovaSeq 6000 S2/S4 reagent kit, v1.5, 300 cycles), generating a raw read count of a minimum of 34 M reads per sample.

Further processing of the raw data was then performed using an in-house pipeline. For comparison, the RNA sequencing data (GSE89189) from Abud et al.^28^ and the dataset (GSE85839) from Muffat et al.^29^ were downloaded and processed in parallel. Quality control of fastq files was performed using FastQC (https://www.bioinformatics.babraham.ac.uk/projects/fastqc/) and MultiQC^42^. Paired-end reads were mapped to the human GRCh38.p13 reference genome (https://www.gencodegenes.org) using HISAT2 v2.2.1^43^. Mapping quality control was done using SAMtools^44^ and Picard (http://broadinstitute.github.io/picard/) metrics. The counts table was obtained using FeatureCounts v2.0.1^45^. Normalization of counts and differential expression analysis for the comparison of pMGL and co-pMG was performed using DESeq2 v1.28.1^46^ in RStudio 1.4.1103, including the biological gender in the model and with the Benjamini–Hochberg method for multiple testing correction. Exploratory data analysis was performed following variance-stabilizing transformation of the counts table, using heat maps and hierarchical clustering with the pheatmap 1.0.12 package (https://github.com/raivokolde/pheatmap) and principal component analysis. Log_2_ fold change (log_2_ fc) shrinkage for the comparison of pMGL and co-pMG was performed using the ashr package v2.2-47^47^. Genes with |log_2_ fc| > 2 and adjusted *p* value < 0.01 were defined as differentially expressed and interpreted with annotations from the Gene Ontology database using clusterProfiler v3.16.1^48^ to perform over-representation analyses.

### Reverse transcription and RT-qPCR

Equal amounts of RNA (30 ng) were reverse-transcribed to cDNA using the High-Capacity cDNA Reverse Transcription Kit (ThermoFisher) according to the manufacturer’s instructions. Quantitative real-time PCR was performed with Fast SYBR™ Green Master Mix (ThermoFisher) according to the manufacturer’s instructions using a LightCycler^®^ 480 PCR System (Roche). The following primers (ChAT from Eurofins Genomics, all others from ThermoFisher) were used:

**Table:** 

Gene	Forward primer sequence	Reverse primer sequence
*C1QA*	GTGACACATGCTCTAAGAAG	GACTCTTAAGCACTGGATTG
*GPR34*	GAAGACAATGAGAAGTCATACC	TGTTGCTGAGAAGTTTTGTG
*P2RY12*	AAGAGCACTCAAGACTTTAC	GGGTTTGAATGTATCCAGTAAG
*TMEM119*	AGTCCTGTACGCCAAGGAAC	GCAGCAACAGAAGGATGAGG
*MERTK*	AGGACTTCCTCACTTTACTAAG	TGAACCCAGAAAATGTTGAC
*ChAT*	CGTGGACAACATCAGATCG	ATGGCCATGACTGTGTATGC
*PROS1*	AAAGATGTGGATGAATGCTC	TCACATTCAAAATCTCCTGG
*GAS6*	CGAAGAAACTCAAGAAGCAG	AGACCTTGATCTCCATTAGG
*TREM2*	TCTGAGAGCTTCGAGGATGC	GGGGATTTCTCCTTCAAGA
*GAPDH*	CTGGGCTACACTGAGCACC	AAGTGGTCGTTGAGGGCAATG

Quantification of the relative fold gene expression of samples was performed using the 2^–∆∆Ct^ method with normalization to the *GAPDH* reference gene.

### Live-imaging of microglial movement and phagocytosis

AH016-3 Lenti-IP-RFP-microglia were co-cultured with healthy control motor neurons in PEI- and Geltrex™-coated glass bottom dishes for confocal microscopy (VWR). The RFP signal was used to identify microglia in co-culture. To visualize microglial movement, images of the RFP signal and brightfield were taken every ~ 30 s for 1 h (2 × 2 stitched images, 20 × magnification) using a Cell Observer spinning disc confocal microscope (Zeiss) equipped with an incubation system (37 °C, 5% CO_2_). To image phagocytic activity, co-cultures were rinsed with Live Cell Imaging Solution (1X, ThermoFisher), and pHrodo™ Green Zymosan Bioparticles™ Conjugates (P35365, ThermoFisher) diluted in Live Cell Imaging Solution (50 µg/mL), which become fluorescent upon phagocytic uptake, were added. The dish was immediately transferred to the spinning disc confocal microscope, and stitched images (3 × 3, 20 × magnification) were acquired every 5 min for 2 h.

### Stimulation with ‘M1’/’M2’ phenotype-inducing agents

To induce pro-inflammatory (‘M1’) or anti-inflammatory (‘M2’) microglial phenotypes, cells were treated with Lipopolysaccharides (LPS, 100 ng/mL, Sigma) and Interferon-γ (IFN-γ, 100 ng/mL, ThermoFisher), or Interleukin-4 (IL-4, 40 ng/mL, R&D Systems) and Interleukin-13 (IL-13, 20 ng/mL, Peprotech), respectively, for 18 h. Vehicle-treated (co-culture medium) cells were used as an unstimulated (‘M0’) control.

### Analysis of microglia clustering

To analyze the clustering of microglia upon pro-inflammatory and anti-inflammatory stimulation, RFP-positive microglia were imaged directly before the addition of ‘M1’/’M2’ inducing agents, and at 9 h and 18 h post-stimulation using the Cell Observer spinning disc confocal microscope (5 × 5 stitched images, 10 × magnification). The number of individual microglial cells and size of microglial clusters was quantified using the “analyze particle” function in Fiji.

### Cytokine/chemokine release measurements

After stimulation with ‘M1’/’M2’-inducing agents, culture supernatants were collected and spun down at 1200 × g for 10 min at 4 °C. Pooled samples from three different healthy control lines for each cell type were analyzed using the Proteome Profiler Human XL Cytokine Array Kit (R&D Systems) according to the manufacturer’s instructions. The signal was visualized on a ChemiDoc™ MP imaging system (Bio-Rad) and analyzed using ImageStudioLite v5.2.5 (LI-COR). Data was then plotted as arbitrary units using the pheatmap 1.0.12 package in RStudio 1.4.1103.

In addition, to confirm the relative expression of Serpin E1 and CHI3L1 in cell culture supernatants, the Human Human Chitinase 3-like 1 Quantikine ELISA Kit (DC3L10) and Human Serpin E1/PAI-1 Quantikine ELISA Kit (DSE100, both R&D Systems) were used according to the manufacturer’s instructions.

### Calcium imaging

pNeuron, pMGL and co-cultures were plated and maintained in WillCo-dish^®^ Glass Bottom Dishes (WillCo Wells) for 14 days. Calcium transients were measured using the fluorescent probe Fluo 4-AM according to the manufacturer’s instructions (ThermoFisher). Cells were incubated with 20 μM Fluo 4-AM resuspended in 0.2% dimethyl sulfoxide for 30 min at RT in Live Imaging Solution (ThermoFisher). After a washing step with Live Imaging Solution, cells were allowed to calibrate at RT for 15–20 min before imaging. Ca^2+^ images were taken by fluorescence microscopy at RT. The dye was excited at 488 nm and images were taken continuously with a baseline recorded for 30 s before stimulation. The stimuli used for calcium release were 50 mM KCl (Sigma-Aldrich) for 30 s, followed by a washing step for one minute. Microglial calcium release was stimulated by 50 µM ADP (Merck) under continuous perfusion for 1 min, followed by a 1-min wash. Analysis of fluorescence intensity was performed using Fiji. Fluorescence measurements are expressed as a ratio (ΔF/F_o_) of the mean change in fluorescence (ΔF) at a pixel relative to the resting fluorescence at that pixel before stimulation (F_o_). The responses were analysed in 20–40 cells per culture.

### Whole-cell patch-clamp electrophysiology

MNs on DIV 33–45 were maintained in a bath temperature of 25 °C in a solution containing 167 mM NaCl, 2.4 mM KCl, 1 mM MgCl_2_, 10 mM glucose, 10 mM HEPES, and 2 mM CaCl_2_ adjusted to a pH of 7.4 and 300 mOsm. Electrodes with tip resistances between 3 and 7 MΩ were produced from borosilicate glass (0.86 mm inner diameter; 1.5 mm outer diameter). The electrode was filled with intracellular solution containing 140 mM K-Gluconate, 6 mM NaCl, 1 mM EGTA, 10 mM HEPES, 4 mM MgATP, 0.5 mM Na_3_GTP, adjusted to pH 7.3 and 290 mOsm. Data acquisition was performed using a Multiclamp 700B amplifier, digidata 1550A and clampEx 6 software (pCLAMP Software suite, Molecular Devices). Data was filtered at 2 kHz and digitized at 10 kHz. Series resistance (R_s_) was continuously monitored and only recordings with stable < 50 MΩ and ΔRs < 20% were included in the analysis. Voltage gated channel currents were measured on voltage clamp, neurons were pre-pulsed for 250 ms with − 140 mV and subsequently a 10 mV-step voltage was applied from − 70 to + 70 mV. Induced action potentials were recorded on current clamp, neurons were held at − 70 mV and 8 voltage steps of 10 mV, from − 10 to 60 mV, were applied. Data was analyzed using Clampfit 10.7 (pCLAMP Software suite).

### Data analysis

Statistical analyses were conducted using GraphPad Prism 9 (GraphPad Software, San Diego, California USA, www.graphpad.com). Comparisons of two groups were performed by two-tailed unpaired t-tests and multiple group comparisons by one-way or two-way analysis of variance (ANOVA) with appropriate post-hoc tests as indicated in the figure legends. The statistical test and number of independent experiments used for each analysis are indicated in each figure legend. Data are presented as single data points and means ± SEM. Differences were considered significant when *P* < 0.05 (*P < 0.05; **P < 0.01; ***P < 0.001; ns: not significant). GraphPad Prism 9 or RStudio 1.4.1103 were used to plot data. Final assembly and preparation of all figures was done using Adobe Illustrator 25.4.1.

## Supplementary Information

Supplementary Video 1.

Supplementary Video 2.

Supplementary Information 1.

## Data Availability

The RNA seq data have been deposited to the GEO repository (GSE200037).

## References

[1] Ilieva H, Polymenidou M, Cleveland DW (2009). Non-cell autonomous toxicity in neurodegenerative disorders: ALS and beyond. J. Cell Biol..

[2] Chen H, Kankel MW, Su SC, Han SWS, Ofengeim D (2018). Exploring the genetics and non-cell autonomous mechanisms underlying ALS/FTLD. Cell Death Differ..

[3] Serio A, Patani R (2018). Concise review: The cellular conspiracy of amyotrophic lateral sclerosis. Stem Cells.

[4] Rossi S, Cozzolino M, Carri MT (2016). Old versus new mechanisms in the pathogenesis of ALS. Brain Pathol..

[5] Vahsen BF (2021). Non-neuronal cells in amyotrophic lateral sclerosis—From pathogenesis to biomarkers. Nat. Rev. Neurol..

[6] Turner MR (2004). Evidence of widespread cerebral microglial activation in amyotrophic lateral sclerosis: An [11C](R)-PK11195 positron emission tomography study. Neurobiol. Dis..

[7] McGeer PL, Itagaki S, McGeer EG (1988). Expression of the histocompatibility glycoprotein HLA-DR in neurological disease. Acta Neuropathol..

[8] Thompson AG (2018). Cerebrospinal fluid macrophage biomarkers in amyotrophic lateral sclerosis. Ann. Neurol..

[9] Thompson AG (2019). CSF chitinase proteins in amyotrophic lateral sclerosis. J. Neurol. Neurosurg. Psychiatry.

[10] Colonna M, Butovsky O (2017). Microglia function in the central nervous system during health and neurodegeneration. Annu. Rev. Immunol..

[11] O'Rourke JG (2016). C9orf72 is required for proper macrophage and microglial function in mice. Science.

[12] Boillee S (2006). Onset and progression in inherited ALS determined by motor neurons and microglia. Science.

[13] Lall D (2021). C9orf72 deficiency promotes microglial-mediated synaptic loss in aging and amyloid accumulation. Neuron.

[14] McCauley ME (2020). C9orf72 in myeloid cells suppresses STING-induced inflammation. Nature.

[15] Wolf SA, Boddeke HW, Kettenmann H (2017). Microglia in physiology and disease. Annu. Rev. Physiol..

[16] Mancuso R (2019). Stem-cell-derived human microglia transplanted in mouse brain to study human disease. Nat. Neurosci..

[17] Giacomelli E (2022). Human stem cell models of neurodegeneration: From basic science of amyotrophic lateral sclerosis to clinical translation. Cell Stem Cell.

[18] Dafinca R (2016). C9orf72 hexanucleotide expansions are associated with altered endoplasmic reticulum calcium homeostasis and stress granule formation in induced pluripotent stem cell-derived neurons from patients with amyotrophic lateral sclerosis and frontotemporal dementia. Stem Cells.

[19] Dafinca R (2020). Impairment of mitochondrial calcium buffering links mutations in C9ORF72 and TARDBP in iPS-derived motor neurons from patients with ALS/FTD. Stem Cell Rep..

[20] van Wilgenburg B, Browne C, Vowles J, Cowley SA (2013). Efficient, long term production of monocyte-derived macrophages from human pluripotent stem cells under partly-defined and fully-defined conditions. PLoS ONE.

[21] Karlsson KR (2008). Homogeneous monocytes and macrophages from human embryonic stem cells following coculture-free differentiation in M-CSF and IL-3. Exp. Hematol..

[22] Buchrieser J, James W, Moore MD (2017). Human induced pluripotent stem cell-derived macrophages share ontogeny with MYB-independent tissue-resident macrophages. Stem Cell Rep..

[23] Monier A (2007). Entry and distribution of microglial cells in human embryonic and fetal cerebral cortex. J. Neuropathol. Exp. Neurol..

[24] Rezaie P, Dean A, Male D, Ulfig N (2005). Microglia in the cerebral wall of the human telencephalon at second trimester. Cereb. Cortex.

[25] Greter M (2012). Stroma-derived interleukin-34 controls the development and maintenance of Langerhans cells and the maintenance of microglia. Immunity.

[26] Haenseler W (2017). A highly efficient human pluripotent stem cell microglia model displays a neuronal-co-culture-specific expression profile and inflammatory response. Stem Cell Rep..

[27] Ababneh NA (2020). Correction of amyotrophic lateral sclerosis related phenotypes in induced pluripotent stem cell-derived motor neurons carrying a hexanucleotide expansion mutation in C9orf72 by CRISPR/Cas9 genome editing using homology-directed repair. Hum. Mol. Genet..

[28] Abud EM (2017). iPSC-derived human microglia-like cells to study neurological diseases. Neuron.

[29] Muffat J (2016). Efficient derivation of microglia-like cells from human pluripotent stem cells. Nat. Med..

[30] Galatro TF (2017). Transcriptomic analysis of purified human cortical microglia reveals age-associated changes. Nat. Neurosci..

[31] Butovsky O (2014). Identification of a unique TGF-beta-dependent molecular and functional signature in microglia. Nat. Neurosci.

[32] Bennett ML (2016). New tools for studying microglia in the mouse and human CNS. Proc. Natl. Acad. Sci. U S A.

[33] Melief J (2012). Phenotyping primary human microglia: Tight regulation of LPS responsiveness. Glia.

[34] Pocock JM, Piers TM (2018). Modelling microglial function with induced pluripotent stem cells: An update. Nat. Rev. Neurosci..

[35] Fernandes HJ (2016). ER stress and autophagic perturbations lead to elevated extracellular alpha-synuclein in GBA-N370S Parkinson's iPSC-derived dopamine neurons. Stem Cell Rep..

[36] Haenseler W (2017). Excess alpha-synuclein compromises phagocytosis in iPSC-derived macrophages. Sci. Rep..

[37] Jonikas M (2018). Stem cell modeling of mitochondrial parkinsonism reveals key functions of OPA1. Ann. Neurol..

[38] Vaughan-Jackson A (2021). Differentiation of human induced pluripotent stem cells to authentic macrophages using a defined, serum-free, open-source medium. Stem Cell Rep..

[39] York EM, LeDue JM, Bernier LP, MacVicar BA (2018). 3DMorph automatic analysis of microglial morphology in three dimensions from ex vivo and in vivo imaging. eNeuro.

[40] Thiry L, Hamel R, Pluchino S, Durcan T, Stifani S (2020). Characterization of human iPSC-derived spinal motor neurons by single-cell RNA sequencing. Neuroscience.

[41] Lamble S (2013). Improved workflows for high throughput library preparation using the transposome-based Nextera system. BMC Biotechnol..

[42] Ewels P, Magnusson M, Lundin S, Kaller M (2016). MultiQC: Summarize analysis results for multiple tools and samples in a single report. Bioinformatics.

[43] Kim D, Paggi JM, Park C, Bennett C, Salzberg SL (2019). Graph-based genome alignment and genotyping with HISAT2 and HISAT-genotype. Nat. Biotechnol..

[44] Li H (2009). The sequence alignment/map format and SAMtools. Bioinformatics.

[45] Liao Y, Smyth GK, Shi W (2014). featureCounts: An efficient general purpose program for assigning sequence reads to genomic features. Bioinformatics.

[46] Love MI, Huber W, Anders S (2014). Moderated estimation of fold change and dispersion for RNA-seq data with DESeq2. Genome Biol..

[47] Stephens M (2017). False discovery rates: A new deal. Biostatistics.

[48] Yu G, Wang LG, Han Y, He QY (2012). clusterProfiler: An R package for comparing biological themes among gene clusters. OMICS.

