# Supplementary material for: Human iPSC co-culture model to investigate the interaction between microglia and motor neurons
Source: Sci Rep. 2022 Jul 23;12:12606. doi: 10.1038/s41598-022-16896-8 (PMC9308778; doi:10.1038/s41598-022-16896-8)
Supplement: Supplementary file 3 — Supplementary Information 1. [file 41598_2022_16896_MOESM3_ESM.pdf]

# SUPPLEMENTARY INFORMATION

**Title:**

**Human iPSC co-culture model to investigate the interaction between microglia and motor neurons**

**Authors:**

Björn F. Vahsen[a, b], Elizabeth Gray[a], Ana Candalija[a], Kaitlyn M. L. Cramb[b, c], Jakub Scaber[a, b], Ruxandra Dafinca[a, b], Antigoni Katsikoudi[b, d], Yinyan Xu[a, b, e], Lucy Farrimond[a, b], Richard Wade-Martins[b, c], William S. James[f], Martin R. Turner[a], Sally A. Cowley[f, *], Kevin Talbot[a, b, *]

**Affiliations:**

[a]Oxford Motor Neuron Disease Centre, Nuffield Department of Clinical Neurosciences, University of Oxford, John Radcliffe Hospital, Oxford OX3 9DU, UK

[b]Kavli Institute for Nanoscience Discovery, University of Oxford, Dorothy Crowfoot Hodgkin Building, South Parks Road, Oxford OX1 3QU, UK

[c]Oxford Parkinson's Disease Centre, Department of Physiology, Anatomy and Genetics, University of Oxford, Dorothy Crowfoot Hodgkin Building, South Parks Road, Oxford OX1 3QX, UK

[d]Molecular Neurodegeneration Research Group, Nuffield Department of Clinical Neurosciences, University of Oxford, Dorothy Crowfoot Hodgkin Building, South Parks Road, Oxford OX1 3QU, UK

[e]Chinese Academy of Medical Sciences (CAMS), CAMS Oxford Institute (COI), Nuffield Department of Medicine, University of Oxford, Oxford OX3 7FZ, UK

[f]James and Lillian Martin Centre for Stem Cell Research, Sir William Dunn School of Pathology, University of Oxford, South Parks Road, Oxford OX1 3RE, UK

*Correspondence: kevin.talbot@ndcn.ox.ac.uk, sally.cowley@path.ox.ac.uk

## SUPPLEMENTARY FIGURES AND LEGENDS

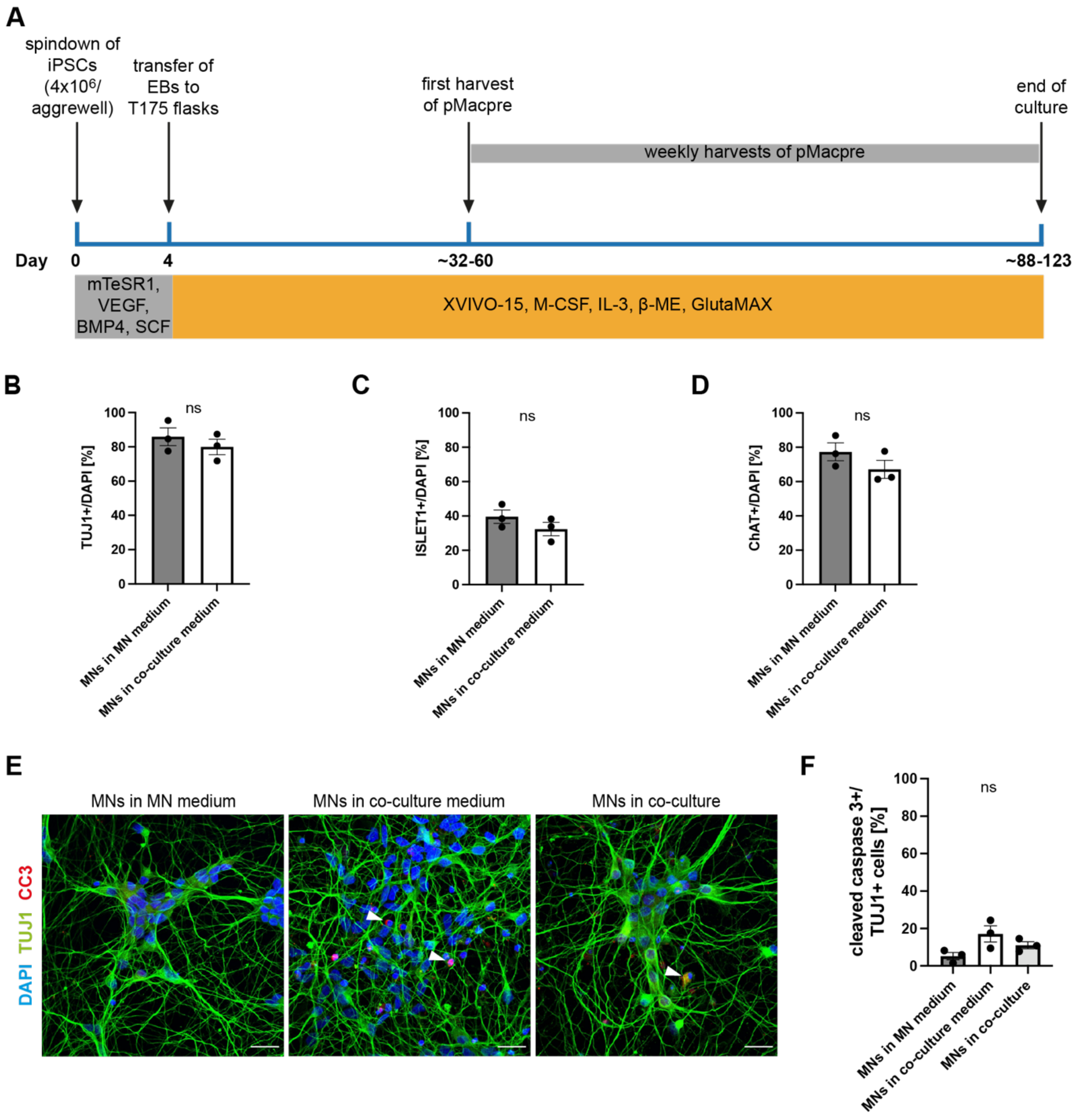


**Supp. Fig. 1: Differentiation protocol for macrophage precursors and complementary assessment of co-culture conditions. A)** Overview of the protocol for the differentiation of iPSC-derived macrophage precursors. **B-D)** Quantification of the expression of the neuron marker TUJ1 (B), and the MN markers ISLET1 (C) and ChAT (D) in MN medium and co-culture medium (n=3 lines from different healthy donors). **E)** Representative images of expression of the apoptosis marker cleaved caspase 3 (CC3) in TUJ1-positive neurons in MN medium (left), co-culture medium (center), and co-culture with microglia (right). White arrowheads indicate CC3-positive cells. Scale bars: 25 µm. **F)** Quantification of CC3 expression in TUJ1-positive neurons in MN medium, co-culture medium, and co-culture with microglia. Single data points from n=3 lines from different healthy donors. Two-tailed unpaired t-test **(B-D)** or one-way ANOVA **(F)**.

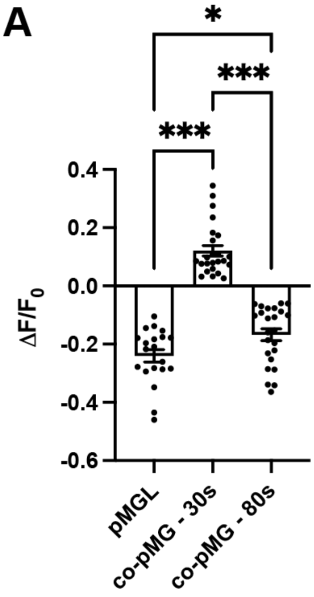


**Supp. Fig. 2: Microglial response in monoculture and co-culture to KCl stimulation in calcium imaging.** pMGL: microglia in monoculture; co-pMG: microglia in co-culture with MNs. **A)** Quantification of calcium transients using the fluorescent probe Fluo 4-AM after stimulation of microglia in monoculture and co-culture with potassium chloride. Single data points represent individual microglial cells from n=1 healthy control line. One-way ANOVA and Tukey's multiple comparisons test.

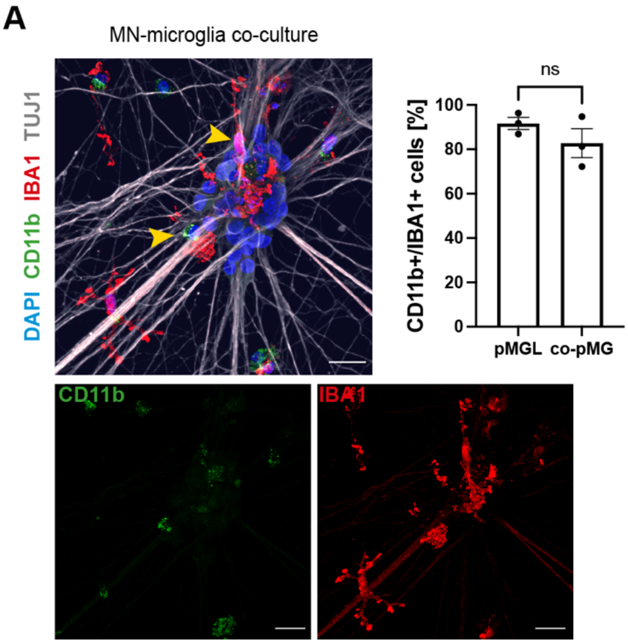


**Supp. Fig. 3: Co-cultured microglia are CD11b-positive. A)** Left: Representative image of iPSC-derived microglia in co-culture with MNs. Yellow arrowheads demonstrate clear co-expression of the macrophage/microglia marker CD11b in IBA1-positive microglia. Scale bars: 25 µm. Right: Quantification of the expression of CD11b in IBA1-positive microglia in monoculture and co-culture with MNs. Single data points from n=3 lines from different healthy donors. Two-tailed unpaired t-test.

**A**

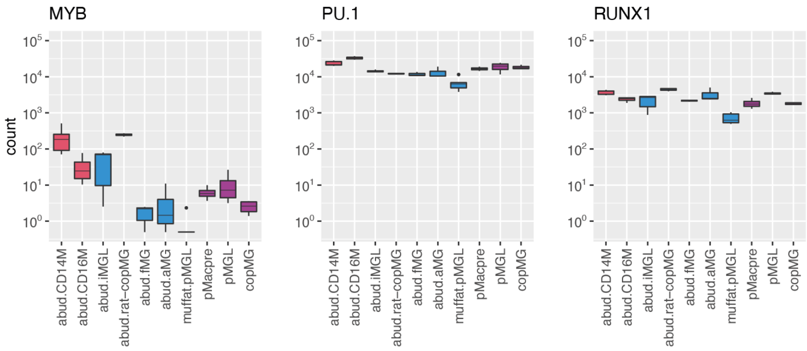


**B**

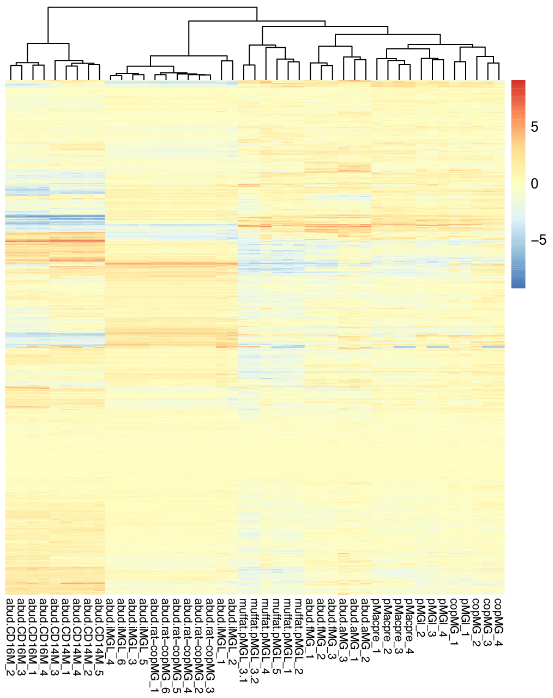


**Supp. Fig. 4: Complementary analysis of transcriptomic data.** Macrophage precursors (pMacpre) and microglia in monoculture (pMGL) and co-culture with MNs (co-pMG) were generated in this study and integrated with samples from [1] (microglia in monoculture: abud.iMGL, microglia in co-culture with rat neurons: abud.rat-copMG, $CD14^+$ blood monocytes: abud.CD14M, $CD16^+$ blood monocytes: abud.CD16M, fetal primary human microglia: abud.fMG, adult primary human microglia: abud.aMG) and [2] (microglia in monoculture: muffat.pMGL). **A)** Box plots of normalized DESeq2 counts for *MYB*, *PU.1*, and *RUNX1* gene expression in RNA sequencing (n=6 samples for abud.iMGL, abud.rat-copMG and muffat.pMGL, n=5 samples for abud.CD16M, n=4 samples for abud.CD14M, pMacpre, pMGL and copMG, n=3 samples for abud.aMG and abud.fMG). **B)** Heatmap of relative VST-transformed values across samples based on set of microglial genes identified by [3] with unsupervised hierarchical clustering between samples using the pheatmap 1.0.12 package.

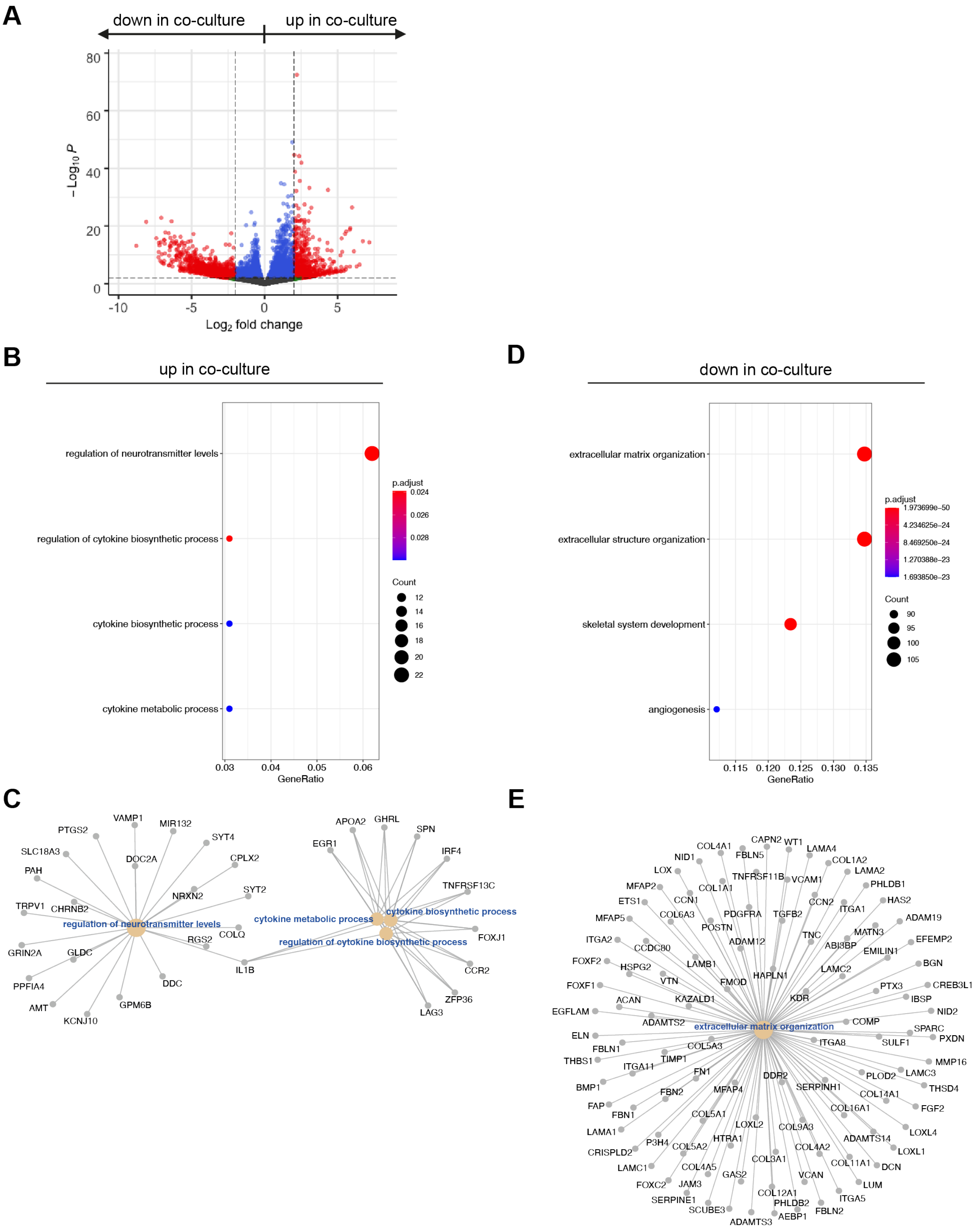


**Supp. Fig. 5: Transcriptomic comparison of microglia in monoculture and co-culture. A)** Volcano plot of all differentially expressed genes between monocultures and co-cultures. Genes with with $| \log_2 fc | > 2$ and adjusted p-value $< 0.01$ were defined as differentially expressed genes (DEGs) and are depicted in red. Up-regulated DEGs: 726, down-regulated DEGs: 843. **B)** Dot plot showing top 4 GO enrichment terms for genes up-regulated in co-culture. **C)** Cnet plot showing DEGs associated with the enriched GO terms in B. **D)** Dot plot showing top 4 GO enrichment terms for genes down-regulated in co-culture. **E)** Cnet plot showing DEGs associated with the enriched GO term “extracellular matrix organization”.

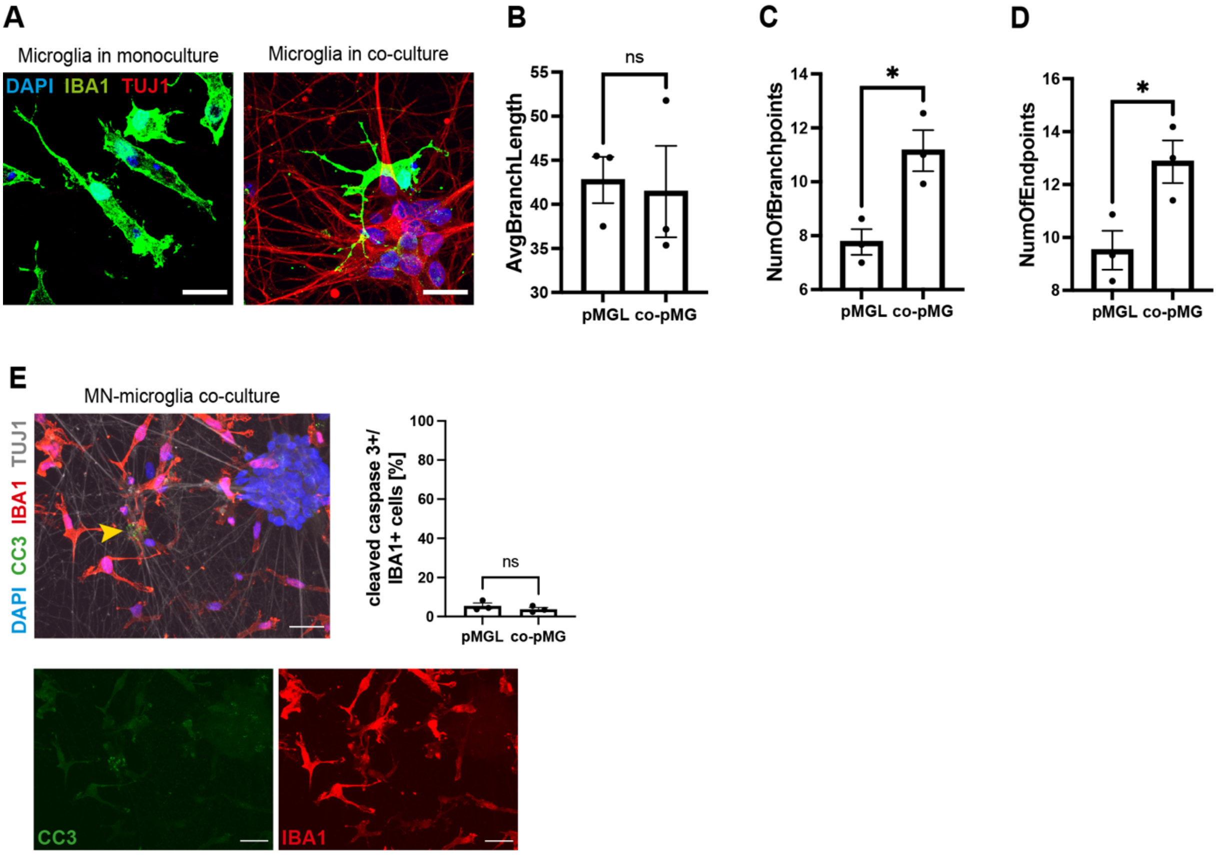


**Supp. Fig. 6: Co-cultured microglia show an enhanced ramified state and minimal cleaved caspase 3 expression. A)** Representative images of microglial ramifications in monoculture (left) and co-culture with MNs (right). Scale bars: 20 µm. **B-D)** Quantification of the average branch length (B), number of branch points (C), and number of end points (D) for microglia in monoculture and co-culture demonstrates significantly enhanced ramifications in co-culture (n=3 lines from different healthy donors). **E)** Left: Representative image of expression of the apoptosis marker cleaved caspase 3 (CC3) in IBA1-positive microglia in co-culture with MNs. Right: Quantification shows low CC3 expression in IBA1-positive microglia in monoculture and co-culture (n=3 lines from different healthy donors). Scale bars: 25 µm. Two-tailed unpaired t-tests.

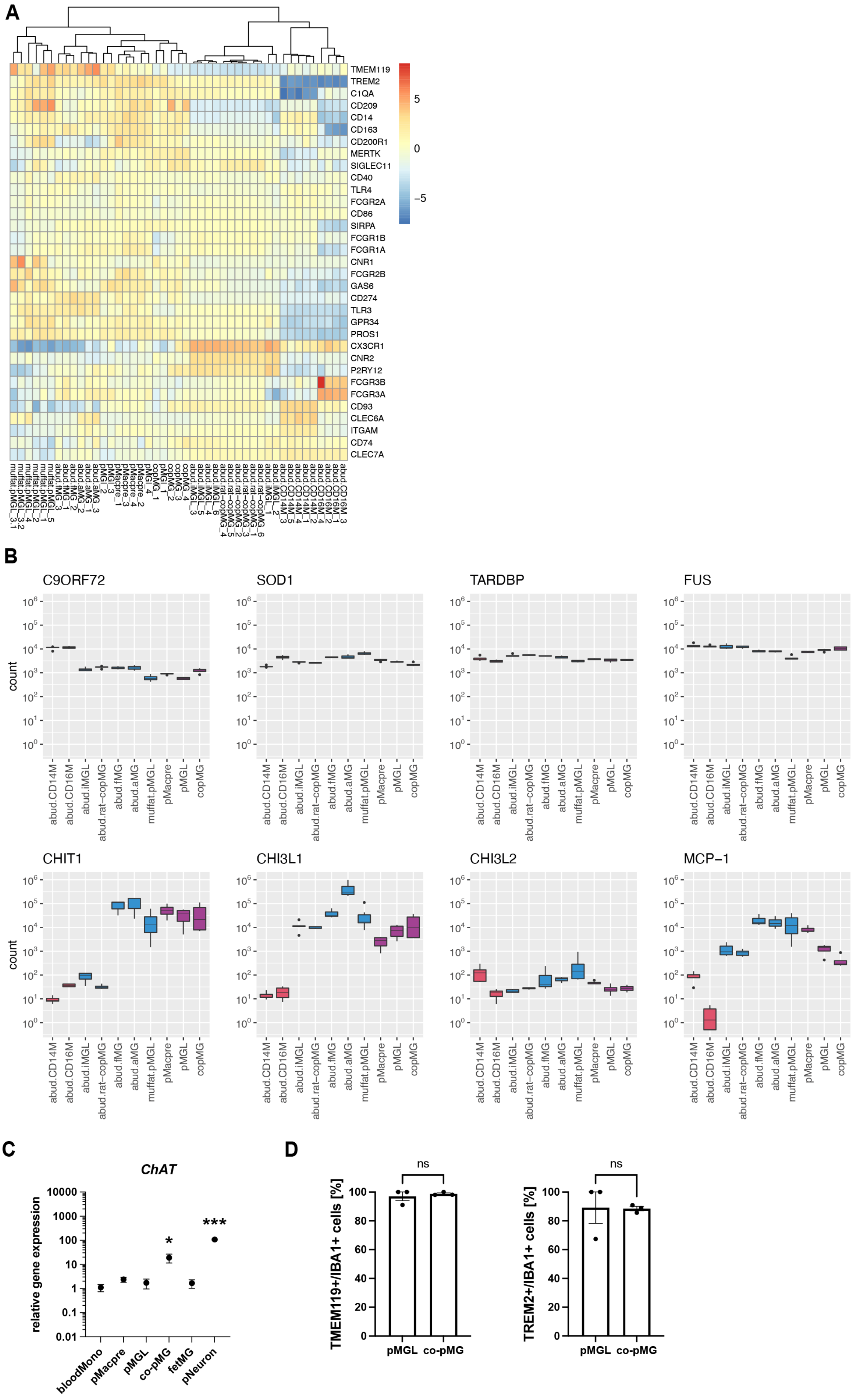
A
TMEM119
TREM2
C1QA
CD209
CD14
CD163
CD200R1
MERTK
SIGLEC11
CD40
TLR4
FCGR2A
CD86
SIRPA
FCGR1B
FCGR1A
CNR1
FCGR2B
GAS6
CD274
TLR3
GPR34
PROS1
CX3CR1
CNR2
P2RY12
FCGR3B
FCGR3A
CD93
CLEC6A
ITGAM
CD74
CLEC7A
5
0
−5
B
C9ORF72
SOD1
TARDBP
FUS
CHIT1
CHI3L1
CHI3L2
MCP−1
count
abud.CD14M
abud.CD16M
abud.iMGL
abud.rat−copMG
abud.fMG
abud.aMG
muffat.pMGL
pMacpre
pMGL
copMG
C
ChAT
relative gene expression
bloodMono
pMacpre
pMGL
co-pMG
fetMG
pNeuron
*
***
D
ns
TMEM119+/IBA1+ cells [%]
TREM2+/IBA1+ cells [%]
pMGL
co-pMG

**Supp. Fig. 7: Complementary analysis of microglia markers and ALS-associated genes. A)** Heatmap of relative VST-transformed values across samples based on microglial genes identified by [4-6] with unsupervised hierarchical clustering between samples. **B)** Box plots of normalized DESeq2 counts for 8 ALS-associated genes in RNA sequencing (n=6 samples for abud.iMGL, abud.rat-copMG and muffat.pMGL, n=5 samples for abud.CD16M, n=4 samples for abud.CD14M, pMacpre, pMGL and copMG, n=3 samples for abud.aMG and abud.fMG). **C)** Quantification of the relative gene expression of the MN marker *ChAT* by RT-qPCR in iPSC-derived macrophage precursors (pMacpre), microglia in monoculture (pMGL), microglia from co-culture after CD11b-MACS (co-pMG), motor neurons in monoculture differentiated in co-culture medium (pNeuron) (all n=3 lines from different healthy donors), human fetal microglia (fetMG, n=3 donors), and human blood monocytes (bloodMono, n=3 healthy patients). Relative expression was calculated using the $2^{-\Delta\Delta Ct}$ method, with GAPDH as an endogenous control and normalization to bloodMono. **D)** Quantification of the expression of TMEM119 and TREM2 in IBA1-positive microglia in monoculture and co-culture with MNs. Single data points from n=3 lines from different healthy donors. One-way ANOVA and Dunnett's multiple comparisons test **(C),** two-tailed unpaired t-test **(D)**.

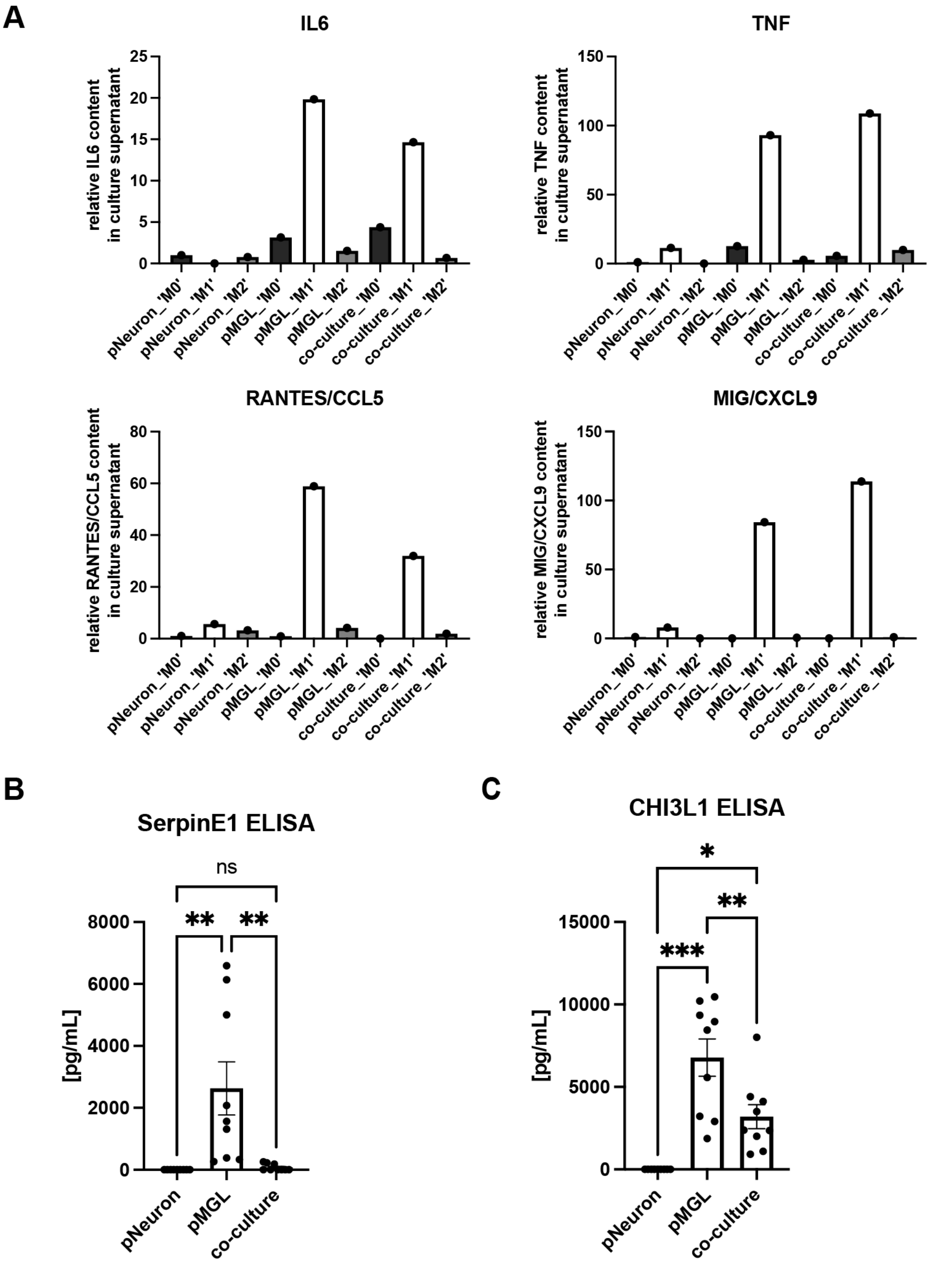


**Supp. Fig. 8: Complementary analysis of the release of cytokines and chemokines from microglia in monoculture and co-culture. A)** Relative content of IL6, TNF, RANTES/CCL5, and MIG/CXCL9 in the culture supernatant determined by Proteome Profiler Human XL Cytokine Array Kit in MNs in monoculture (pNeuron), microglia in monoculture (pMGL), and co-culture. Samples from n=3 lines from different healthy donors were pooled for each condition, either treated with vehicle ('M0'), LPS/IFN-γ ('M1'), or IL-4/IL-13 ('M2') for 18 h. **B)** Quantification of Serpin E1 release into the culture supernatant by ELISA in pNeuron, pMGL, and co-culture. Pooled samples either treated with vehicle ('M0'), LPS/IFN-γ ('M1'), or IL-4/IL-13 ('M2') for 18 h (all n=3 lines from different healthy donors). **C)** Quantification of CHI3L1 release into the culture supernatant by ELISA in pNeuron, pMGL, and co-culture, pooled samples either treated with vehicle ('M0'), LPS/IFN-γ ('M1'), or IL-4/IL-13 ('M2')

for 18 h (all n=3 lines from different healthy donors). One-way ANOVA and Tukey's multiple comparisons test.

## SUPPLEMENTARY TABLES AND LEGENDS

**Supp. Table 1:** Cytokine release into the culture supernatant from MNs in monoculture (pNeuron), microglia in monoculture (pMGL), and co-cultures was measured using the Proteome Profiler Human XL Cytokine Array Kit. Cells were either treated with vehicle ('M0'), LPS/IFN-γ ('M1'), or IL-4/IL-13 ('M2') for 18 h. Pooled supernatant from n=3 lines from different healthy donors was used for each condition. Data represent the mean of two technical replicates per condition. Values correspond to the signal intensity value expressed as arbitrary units.

| Released factor | pNeu-ron 'M0' | pNeu-ron 'M1' | pNeu-ron 'M2' | pMGL 'M0' | pMGL 'M1' | pMGL 'M2' | co-culture 'M0' | co-culture 'M1' | co-culture 'M2' |
|---|---|---|---|---|---|---|---|---|---|
| Adiponectin | 207 | 414 | 458 | 365 | 637 | 421 | 396 | 388 | 214 |
| Apolipoprotein A-I | 360 | 655 | 566 | 486 | 1446 | 1017 | 1170 | 1003 | 431 |
| Angiogenin | 3865 | 2930 | 4653 | 403 | 461 | 395 | 3675 | 3268 | 4058 |
| Angiopoietin-1 | 0 | 0 | 350 | 0 | 0 | 0 | 31 | 0 | 170 |
| Angiopoietin-2 | 261 | 393 | 746 | 213 | 457 | 498 | 107 | 474 | 66 |
| BAFF | 312 | 165 | 186 | 144 | 173 | 143 | 133 | 93 | 274 |
| BDNF | 954 | 1509 | 1384 | 14580 | 10012 | 9560 | 1611 | 1179 | 1529 |
| C5/C5a | 0 | 286 | 110 | 327 | 469 | 374 | 15 | 106 | 0 |
| CD14 | 0 | 0 | 0 | 2556 | 1760 | 1220 | 1000 | 1303 | 707 |
| CD30 | 0 | 131 | 96 | 245 | 175 | 193 | 54 | 302 | 0 |
| CD40 ligand | 0 | 136 | 0 | 0 | 357 | 0 | 341 | 520 | 0 |
| Chitinase 3-like 1 | 217 | 295 | 533 | 11775 | 15954 | 14700 | 11445 | 8735 | 8309 |
| Complement Factor D | 26 | 131 | 0 | 437 | 711 | 563 | 269 | 85 | 0 |
| C-Reactive Protein | 0 | 0 | 0 | 0 | 496 | 260 | 190 | 225 | 0 |
| Cripto-1 | 0 | 0 | 123 | 147 | 216 | 0 | 99 | 103 | 5 |
| Cystatin C | 0 | 194 | 252 | 336 | 186 | 74 | 762 | 381 | 654 |
| Dkk-1 | 587 | 212 | 0 | 1562 | 743 | 394 | 0 | 30 | 452 |
| DPPIV | 191 | 0 | 212 | 3354 | 5264 | 1914 | 1145 | 1580 | 1265 |
| EGF | 0 | 0 | 204 | 0 | 0 | 0 | 125 | 0 | 83 |
| Emmprin | 169 | 642 | 740 | 239 | 172 | 160 | 775 | 1354 | 1000 |
| ENA-78 | 1300 | 1220 | 2212 | 13446 | 2721 | 4223 | 9715 | 13820 | 5257 |
| Endoglin | 37 | 0 | 162 | 868 | 809 | 380 | 429 | 419 | 232 |
| Fas Ligand | 0 | 163 | 282 | 389 | 321 | 0 | 0 | 111 | 155 |
| FGF basic | 240 | 0 | 102 | 150 | 599 | 93 | 150 | 213 | 65 |
| FGF-7 | 0 | 71 | 0 | 0 | 407 | 0 | 0 | 0 | 160 |
| FGF-19 | 2633 | 1979 | 2425 | 931 | 2188 | 1214 | 3226 | 2537 | 3197 |
| Flt-3 Ligand | 0 | 0 | 0 | 98 | 0 | 83 | 57 | 0 | 0 |
| G-CSF | 0 | 0 | 194 | 246 | 298 | 0 | 38 | 139 | 115 |
| GDF-15 | 2096 | 1978 | 2276 | 1018 | 799 | 180 | 1542 | 2699 | 3290 |
| GM-CSF | 146 | 77 | 0 | 409 | 0 | 0 | 0 | 0 | 187 |
| GROα | 103 | 156 | 1159 | 5065 | 5649 | 2510 | 4305 | 6851 | 2906 |
| Growth Hormone | 147 | 0 | 213 | 0 | 0 | 274 | 0 | 0 | 0 |
| HGF | 0 | 0 | 0 | 196 | 365 | 204 | 0 | 169 | 354 |
| ICAM-1 | 0 | 144 | 245 | 485 | 1287 | 1586 | 2122 | 1741 | 1181 |
| IFN-γ | 0 | 2447 | 0 | 215 | 3763 | 437 | 87 | 3576 | 344 |
| IGFBP-2 | 9506 | 8742 | 11120 | 783 | 963 | 722 | 9597 | 8964 | 10640 |

| | | | | | | | | | |
|---|---|---|---|---|---|---|---|---|---|
| IGFBP-3 | 121 | 0 | 160 | 219 | 187 | 87 | 0 | 67 | 166 |
| IL-1α | 270 | 0 | 355 | 203 | 364 | 109 | 183 | 415 | 85 |
| IL-1β | 0 | 520 | 43 | 224 | 231 | 232 | 44 | 133 | 12 |
| IL-1ra | 412 | 0 | 257 | 508 | 707 | 1360 | 267 | 1084 | 1330 |
| IL-2 | 0 | 0 | 0 | 218 | 46 | 0 | 16 | 0 | 175 |
| IL-3 | 0 | 64 | 0 | 422 | 0 | 44 | 17 | 371 | 174 |
| IL-4 | 0 | 82 | 15209 | 612 | 720 | 13052 | 519 | 0 | 14173 |
| IL-5 | 377 | 303 | 186 | 61 | 36 | 410 | 0 | 0 | 0 |
| IL-6 | 365 | 1 | 287 | 1150 | 7251 | 560 | 1601 | 5357 | 245 |
| IL-8 | 1528 | 1218 | 1304 | 22259 | 21211 | 19849 | 21561 | 20167 | 21654 |
| IL-10 | 0 | 94 | 63 | 82 | 557 | 226 | 238 | 73 | 0 |
| IL-11 | 681 | 709 | 518 | 0 | 429 | 0 | 183 | 689 | 862 |
| IL-12 p70 | 209 | 203 | 0 | 0 | 180 | 215 | 102 | 947 | 0 |
| IL-13 | 0 | 0 | 3598 | 0 | 101 | 1806 | 0 | 233 | 2089 |
| IL-15 | 156 | 212 | 95 | 134 | 25 | 21 | 0 | 0 | 330 |
| IL-16 | 287 | 1000 | 0 | 0 | 953 | 98 | 13 | 1042 | 12 |
| IL-17A | 326 | 413 | 450 | 1586 | 2275 | 300 | 630 | 629 | 937 |
| IL-18 Bpa | 0 | 11319 | 516 | 204 | 2799 | 258 | 280 | 10248 | 43 |
| IL-19 | 49 | 93 | 354 | 0 | 0 | 871 | 0 | 500 | 848 |
| IL-22 | 0 | 262 | 0 | 343 | 344 | 167 | 250 | 367 | 56 |
| IL-23 | 75 | 10 | 251 | 0 | 0 | 149 | 72 | 0 | 37 |
| IL-24 | 53 | 0 | 167 | 73 | 0 | 0 | 0 | 0 | 614 |
| IL-27 | 565 | 0 | 74 | 0 | 94 | 0 | 254 | 410 | 111 |
| IL-31 | 73 | 211 | 260 | 7 | 0 | 252 | 28 | 333 | 198 |
| IL-32 | 113 | 0 | 86 | 0 | 0 | 166 | 0 | 99 | 0 |
| IL-33 | 0 | 291 | 0 | 0 | 614 | 0 | 0 | 115 | 312 |
| IL-34 | 441 | 125 | 0 | 522 | 0 | 333 | 566 | 0 | 28 |
| IP-10 | 66 | 27437 | 159 | 0 | 23231 | 48 | 628 | 24683 | 502 |
| I-TAC | 0 | 0 | 0 | 100 | 21155 | 0 | 233 | 21787 | 28 |
| Kallikrein 3 | 0 | 8 | 65 | 140 | 475 | 0 | 107 | 0 | 0 |
| Leptin | 209 | 0 | 225 | 34 | 397 | 4 | 128 | 711 | 0 |
| LIF | 53 | 0 | 0 | 218 | 149 | 150 | 142 | 105 | 0 |
| Lipocalin-2 | 277 | 0 | 132 | 132 | 541 | 141 | 33 | 220 | 451 |
| MCP-1 | 5111 | 8098 | 5527 | 12681 | 12693 | 9241 | 11796 | 12401 | 12463 |
| MCP-3 | 0 | 204 | 0 | 1651 | 5882 | 422 | 0 | 1569 | 264 |
| M-CSF | 27 | 0 | 117 | 134 | 384 | 29 | 1 | 92 | 132 |
| MIF | 3619 | 2824 | 5371 | 906 | 502 | 687 | 3723 | 3595 | 3355 |
| MIG | 160 | 1273 | 0 | 0 | 13537 | 81 | 0 | 18264 | 156 |
| MIP-1α/MIP-1β | 79 | 40 | 149 | 12030 | 10909 | 6349 | 9201 | 11876 | 1047 |
| MIP-3α | 419 | 734 | 327 | 1455 | 1862 | 106 | 2468 | 8707 | 155 |
| MIP-3β | 347 | 81 | 313 | 324 | 2312 | 0 | 382 | 2392 | 105 |
| MMP-9 | 32 | 180 | 158 | 6299 | 1796 | 3174 | 1940 | 509 | 2119 |
| Myeloperoxidase | 0 | 0 | 0 | 0 | 648 | 169 | 0 | 0 | 0 |
| Osteopontin | 11996 | 13209 | 13726 | 13751 | 6450 | 6056 | 13650 | 13829 | 14835 |
| PDGF-AA | 2090 | 1947 | 1660 | 735 | 807 | 176 | 2497 | 1912 | 2816 |
| PDGF-AB/BB | 0 | 0 | 201 | 162 | 338 | 19 | 0 | 0 | 0 |
| Pentraxin 3 | 1672 | 1759 | 2629 | 4441 | 2972 | 1676 | 3116 | 4039 | 3122 |
| PF4 | 0 | 0 | 166 | 86 | 0 | 200 | 0 | 0 | 59 |
| RAGE | 227 | 61 | 0 | 221 | 3 | 126 | 0 | 172 | 0 |
| RANTES | 56 | 317 | 183 | 50 | 3328 | 236 | 0 | 1807 | 105 |
| RBP-4 | 0 | 39 | 357 | 0 | 0 | 85 | 0 | 0 | 230 |
| Relaxin-2 | 0 | 38 | 223 | 31 | 42 | 0 | 10 | 117 | 55 |
| Resistin | 326 | 320 | 357 | 440 | 575 | 387 | 122 | 467 | 448 |
| SDF-1α | 74 | 272 | 539 | 1319 | 1462 | 925 | 866 | 967 | 737 |

| | | | | | | | | | |
|---|---|---|---|---|---|---|---|---|---|
| Serpin E1 | 6760 | 5777 | 4827 | 25167 | 22004 | 25232 | 10719 | 9587 | 11453 |
| SHBG | 90 | 0 | 0 | 877 | 918 | 963 | 0 | 0 | 0 |
| ST2 | 0 | 0 | 84 | 316 | 423 | 185 | 0 | 0 | 0 |
| TARC | 0 | 0 | 0 | 243 | 248 | 1349 | 107 | 269 | 9382 |
| TFF3 | 2275 | 2009 | 4218 | 44 | 290 | 101 | 1134 | 1484 | 1397 |
| TfR | 0 | 59 | 0 | 291 | 203 | 342 | 0 | 0 | 0 |
| TGF-α | 217 | 0 | 105 | 148 | 234 | 120 | 22 | 136 | 5 |
| Thrombospon din-1 | 0 | 128 | 0 | 3173 | 2060 | 2241 | 387 | 438 | 368 |
| TNF-α | 38 | 439 | 0 | 488 | 3585 | 106 | 216 | 4192 | 381 |
| uPAR | 0 | 0 | 0 | 2196 | 1487 | 427 | 534 | 369 | 443 |
| VEGF | 1439 | 1242 | 1408 | 195 | 0 | 37 | 1128 | 1032 | 1343 |
| Vitamin D BP | 259 | 138 | 164 | 156 | 674 | 456 | 189 | 239 | 88 |
| CD31 | 285 | 0 | 368 | 394 | 382 | 279 | 142 | 367 | 999 |
| TIM-3 | 0 | 165 | 221 | 1177 | 1994 | 307 | 528 | 757 | 516 |
| VCAM-1 | 415 | 997 | 94 | 1329 | 2329 | 540 | 2146 | 7716 | 1468 |

## SUPPLEMENTARY VIDEOS AND LEGENDS

**Movie S1: Co-cultured microglia have highly dynamic ramifications.** Images were taken every ~30 s for 10 min.

**Movie S2: Co-cultured microglia take up pHrodo zymosan particles.** Images were taken every 5 min for 2 h.

## REFERENCES


1 Abud, E. M. *et al.* iPSC-Derived Human Microglia-like Cells to Study Neurological Diseases. *Neuron* **94**, 278-293 e279, doi:10.1016/j.neuron.2017.03.042 (2017).

2 Muffat, J. *et al.* Efficient derivation of microglia-like cells from human pluripotent stem cells. *Nat Med* **22**, 1358-1367, doi:10.1038/nm.4189 (2016).

3 Galatro, T. F. *et al.* Transcriptomic analysis of purified human cortical microglia reveals age-associated changes. *Nat Neurosci* **20**, 1162-1171, doi:10.1038/nn.4597 (2017).

4 Butovsky, O. *et al.* Identification of a unique TGF-beta-dependent molecular and functional signature in microglia. *Nat Neurosci* **17**, 131-143, doi:10.1038/nn.3599 (2014).

5 Bennett, M. L. *et al.* New tools for studying microglia in the mouse and human CNS. *Proc Natl Acad Sci U S A* **113**, E1738-1746, doi:10.1073/pnas.1525528113 (2016).

6 Melief, J. *et al.* Phenotyping primary human microglia: tight regulation of LPS responsiveness. *Glia* **60**, 1506-1517, doi:10.1002/glia.22370 (2012).
